# Fully automated deep learning models with smartphone applicability for prediction of pain using the Feline Grimace Scale

**DOI:** 10.1038/s41598-023-49031-2

**Published:** 2023-12-07

**Authors:** P. V. Steagall, B. P. Monteiro, S. Marangoni, M. Moussa, M. Sautié

**Affiliations:** 1 Department of Clinical Sciences, Faculty of Veterinary Medicine, Université de Montréal, Saint-Hyacinthe, QC Canada; 2 Department of Veterinary Clinical Sciences and Centre for Animal Health and Welfare, Jockey Club College of Veterinary Medicine and Life Sciences, City University of Hong Kong, Hong Kong, China; 3 Plateforme IA-Agrosanté, Université de Montréal, Saint-Hyacinthe, QC Canada

**Keywords:** Neuroscience, Medical research

## Abstract

This study used deep neural networks and machine learning models to predict facial landmark positions and pain scores using the Feline Grimace Scale^©^ (FGS). A total of 3447 face images of cats were annotated with 37 landmarks. Convolutional neural networks (CNN) were trained and selected according to size, prediction time, predictive performance (normalized root mean squared error, NRMSE) and suitability for smartphone technology. Geometric descriptors (n = 35) were computed. XGBoost models were trained and selected according to predictive performance (accuracy; mean square error, MSE). For prediction of facial landmarks, the best CNN model had NRMSE of 16.76% (ShuffleNetV2). For prediction of FGS scores, the best XGBoost model had accuracy of 95.5% and MSE of 0.0096. Models showed excellent predictive performance and accuracy to discriminate painful and non-painful cats. This technology can now be used for the development of an automated, smartphone application for acute pain assessment in cats.

## Introduction

Recognition of pain is the first step for appropriate treatment and essential to understand if analgesic therapies provide adequate pain relief^1^. In individuals that cannot self-report, pain assessment is challenging and commonly relies on evoked behavioral responses or the use of pain scoring systems^2^. However, these methods may lack validity, be cumbersome, observer-, training-, time- and gender-dependent and may not always capture the affective and motivational complexity of pain. Indeed, pain management is crucial to obtain reliable research outcomes in biomedical and neuroscience research using laboratory animals^3^. Additionally, the benefits of pet ownership and the human-animal bond are undeniable to our society^4^, especially after the COVID-19 pandemic^5^ and for children^6^ as well as for the use of naturally occurring disease models for translational research using domestic species^7^. Therefore, pain assessment is also crucial for veterinary health professionals^8,9^. A potential solution to overcome these aforementioned challenges is the use of technologies for automated pain assessment using artificial intelligence^10,11^. This approach would eliminate observer bias, the workload (i.e. training) and time required for pain assessment in research. This would be of particular interest for end users and knowledge dissemination if such systems could be integrated into smartphone applications.

Changes in facial expressions related to pain have been identified in many animal species^12,13^. They can be used to discriminate painful and non-painful individuals using grimace scales and scoring of action units (AU) that comprise a facial expression. The detailed applications and limitations of grimace scales are discussed elsewhere^14,15^. However, still image or real-time pain scoring using grimace scales can be labor intensive and again, dependent on several factors^16^, including video- and/or image capture and manual scoring^17^. It is clear that fully automated models for facial recognition and grimace scale scoring are needed in pain research^11,18,19^. Despite some advances in this field, research has not been published with fully automated models that include algorithm outputs of numerical grimace scale scores using dataset of animals of any coat color and type (i.e. short or long haired), breed, gender and age, and with naturally-occurring pain of different conditions (i.e. medical, surgical, trauma, etc.). The automated discrimination of painful and non-painful animals would provide guidance to researchers and veterinarians to the need, or not, of the administration of analgesics without individual bias related to training or gender.

The Feline Grimace Scale^©^ (FGS) is a valid, reliable, simple and practical tool for acute pain assessment in cats. It includes five action units (AU; ear position, orbital tightening, muzzle tension, whiskers change, and head position)^20^. Each AU is scored from 0 to 2, where 0 = AU is absent, 1 = moderate presence of AU or uncertainty over its presence or absence, and 2 = obvious presence of AU. The FGS score is the sum of scores from each AU divided by the maximum possible score; scores ≥ 0.39/1 indicate that the cat is likely in pain requiring intervention. The FGS can be used for any type of acute pain and by veterinary professionals and caregivers^8,21,22^.

This study aimed to use convolutional neural network (CNN) models to predict facial landmark positions and FGS scores^23^. For prediction of landmark positions, models were evaluated regarding predictive performance, model size and prediction time for potential integration into smartphone applications. For prediction of FGS scores, models were evaluated for their discriminatory ability (painful or not painful), accuracy and error. The authors wanted to evaluate model backbones that would be applicable to automated pain assessment in cats but also for other grimace scales in mammalian species.

## Results

### Phase I—Prediction of facial landmark positions

**Figure 1:**
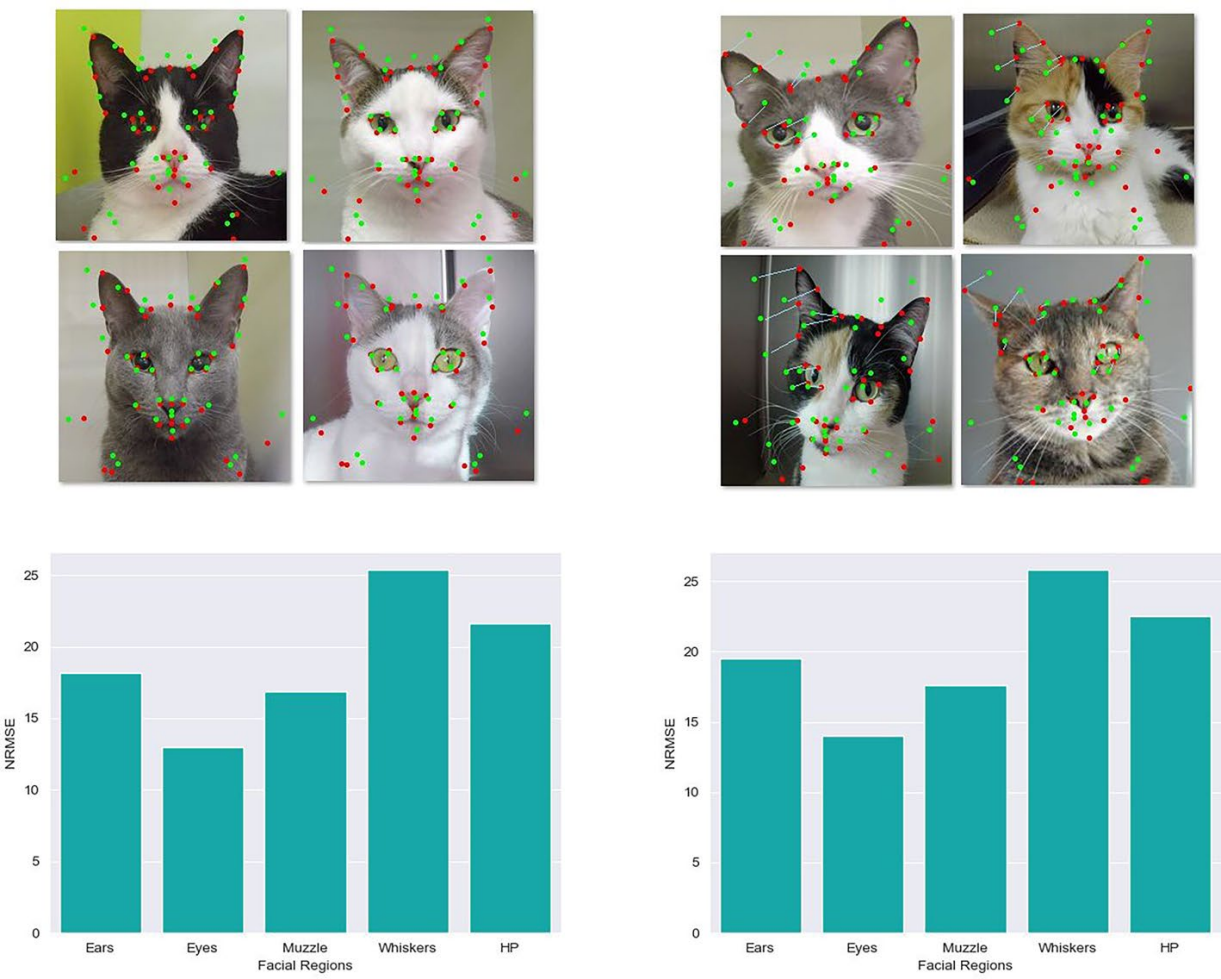
Results from facial landmark prediction. Top: examples of facial images of cats showing predictive performance of two convolutional neural network models (ShuffleNetV2_ 0.75_1) for the prediction of 37 landmarks with (left) and without (right) face alignment. ShuffleNetV2 models were based on the use of pointwise group, depthwise convolutions, bottleneck-like structures, and a channel shuffle operation. The first number after the architecture name is the scale factor (0.75) and the second number is the bottleneck ratio (1). Green dots: predicted landmarks. Red dots: ground truth landmarks. In the images of unaligned faces (top right), the distances between the predicted and ground truth landmarks for one of the ears and one of eyes are illustrated with blue light lines. Both models include preprocessing by Laplacian edge detection filter of kernel size 3 × 3. Preprocessing by face alignment improved predictive performance as observed by the green and red dots becoming closer. Bottom: bar graphs showing the predictive performance for facial landmark positions linked to each of the five action units of the Feline Grimace Scale (ear position, orbital tightening, muzzle tension, whiskers change and head position) with (left) and without (right) face alignment. The models were evaluated on a test dataset of 120 randomly selected images. Data are reported as normalized root mean square error (NRMSE (%)), which were lower when with face alignment indicating a better fit between the predicted and ground truth landmarks (bottom left).

A total of 11 CNN-based models with different architectures and trained on different augmented datasets were selected. A summary of the size, prediction time and predictive performance of these models is presented on Table 1 and Supplementary Figs. S1–S5. Regardless of the proposed transformations, the ShuffleNetV2, EfficientNetB0 and MobileNetV3 architectures showed the best predictive performances (NRMSE of 16.76%, 16.89% and 18.16%, respectively). Face alignment increased the predictive performance and preprocessing times (prediction time), especially when used in conjunction with the Laplacian filter (Fig. 1, Supplementary Fig. S2). The models without any preprocessing edge detection filters showed the lowest predictive performance and largest differences amongst AU for prediction errors. Whiskers change and head position showed the largest prediction errors whereas orbital tightening and muzzle tension, the smallest prediction errors (Fig. 1).

**Table 1:** Model size, prediction time and predictive performance of 11 convolutional neural network models (CNN) analyzed for the automated prediction of 37 facial landmarks using 120 random facial images of cats.

CNN models	Non-aligned faces			Aligned faces			Np (10^6^)	Dataset
	NRMSE (%)	NRMSEw (%)	Time (s/i)	NRMSE (%)	NRMSEw (%)	Time (s/i)		
ShuffleNetV2_0.75_1_F	18.08	17.14	0.0406	16.76	14.85	0.0460	6.17	Lap
EfficientNetB0_F_M	18.68	17.71	0.0687	16.89	15.12	0.0813	10.10	Lap
EfficientNetB0_F	19.33	18.20	0.0625	17.17	15.79	0.0708	8.69	Lap
MobileNetV3Large_1.0_A	20.21	18.77	0.0399	18.16	16.89	0.0472	4.32	Lap
MobileNetV3Large_Min_F	20.44	19.07	0.0335	19.12	17.95	0.0408	2.76	Lap
EfficientNetB0_A	20.75	18.72	0.0483	19.16	18.15	0.0574	4.14	No-Lap
ShuffleNetV2_0.5_1_A_M	22.17	20.59	0.0322	18.67	17.01	0.0409	2.50	No-Lap
ShuffleNetV2_0.5_1_A_S	22.24	21.72	0.0383	18.71	17.42	0.0353	3.93	No-Lap
ShuffleNetV2_0.5_1_F_FC_H	22.56	21.25	0.0407	19.24	18.03	0.0419	10.63	No-Lap
EfficientNetB0_F_FC_H	22.61	21.39	0.0701	19.71	18.12	0.0788	10.18	No-Lap
EfficientNetB0_-41_F	22.79	21.07	0.0558	19.12	18.11	0.0598	6.16	Lap

A: GlobalAveragePooling2D (GAP2D) layer. S: Block of parallel convolutional layers with symmetric kernels. M: Block of parallel convolutional layers with asymmetric kernels. H: Block of parallel convolutional layers with hybrid (symmetric and asymmetric) kernels. F: Flatten layer. FC: Fully Connected Layers. Min: minimalistic version of the corresponding model. The first numbered notations next to the models’ name indicate scale factor (ShuffleNetV2), width multiplier (MobileNetV3Large), or that the model does not have the 41 top layers (EfficientNetB0_-41_F). For ShuffleNetV2 models, the second number indicates the bottleneck ratio.

Prediction time and predictive performance were evaluated for aligned and non-aligned faces. Size was measured as number of parameters and reported as Np × 10^6^. Prediction time was measured as inference plus preprocessing time and reported as seconds per image (s/i). Predictive performance was measured as Normalized Root Mean Squared Error (NRMSE) and reported as percentage (%). The NRMSEw refers to the NRMSE calculated after excluding the 10 landmarks with the highest prediction errors (landmarks 6; 7; 27; 28; 29; 30; 31; 32; 36; 37). The CNN models were trained on two types of datasets including those with or without preprocessing by Laplacian filters (Lap or No-Lap, respectively).

The structural transformations showed that the replacement of the GAP2D layer by a flatten layer increased predictive performance. Symmetric parallel convolutional layer blocks increased the performance in most of the architectures and had a better predictive performance than asymmetric or hybrid kernels.

### Phase II—Prediction of FGS scores

Predictive performances of the models used for binary classification, regression and ordinal classification using different combinations of geometric descriptors or AU are reported in Tables 2, 3 and 4, respectively. For binary classification models, those using the ‘AND’ aggregation function rule and including all 35 geometric descriptors achieved the highest accuracy (95.5%) and AUROC of 0.97 (Supplementary Figs. S6–S7). The hyperparameter "scale_pos_weight" was assigned 4.77 when the output variable was obtained using the AND aggregation function and 2.64 when the OR aggregation function was used. For regression models, those using the ‘Mean’ aggregation function rule and including only geometric descriptors selected by Boruta–Shap algorithm (n = 10) achieved the lowest error (MSE = 0.0096). For ordinal classification models, those using the ‘Mode’ aggregation function rule performed best for most AU, except for whiskers change and head position for which the ‘Minimum’ aggregation performed better. The largest prediction errors were observed for the AU muzzle tension and whiskers change. The PCA scores confirmed the discriminatory ability of binary classification models between ‘painful’ and ‘non-painful’ cats (Supplementary Figs. S8–S9).

**Table 2:** Predictive performance of binary classification models based on Feline Grimace Scale (FGS) and different combinations of geometric descriptors (features).

Features	AND rule			OR rule		
	Accuracy (%)	AUROC	N	Accuracy (%)	AUROC	N
All GD	95.51	0.97	35	92.86	0.95	35
RFE	94.38	0.96	30	93.26	0.94	16
Boruta-Shap	92.46	0.95	10	89.89	0.94	18
wWhiskers	94.05	0.97	30	88.76	0.94	30
wHP	93.26	0.97	28	92.13	0.95	28
wWHP	91.01	0.96	23	89.89	0.93	23

All GD: all 35 geometric descriptors. RFE: geometric descriptors selected by the recursive feature elimination algorithm. Boruta–Shap: geometric descriptors selected by the Boruta algorithm based on Shapley values. wWhiskers: geometric descriptors resulting from the exclusion of those associated with the action unit Whiskers change. wHP: geometric descriptors resulting from the exclusion of those associated with the action unit Head position. wWHP: geometric descriptors resulting from the exclusion of those associated with the action unit Whiskers change and Head position. N: number of geometric descriptors.

Binary classification of ‘painful’ or ‘non-painful’ cats was based on total FGS scores and cut-off scores for administration of analgesia (FGS scores ≥ 0.4/1) as previously reported^20^. Data are reported as accuracy (%) and area under the receiver operator characteristic curve (AUROC). The ‘AND’ and ‘OR’ rules were used to aggregate the values assigned to the same image by multiple raters. For example, using the rule ‘AND’, an image was assigned 1 if and only all raters assigned 1 to that image and 0 if at least one rater assigned 1. Using the rule ‘OR’, an image was assigned 1 if at least one rater assigned 1 to that image and 0 if all raters assigned 0 to that image.

**Table 3:** Predictive performance of regression models based on Feline Grimace Scale (FGS) scores and different combinations of geometric descriptors (features).

Features	Mean		Maximum		Minimum	
	MSE	N	MSE	N	MSE	N
All GD	0.0104	35	0.0129	35	0.0121	35
RFE	0.0101	32	0.0136	26	0.0127	30
Boruta-Shap	0.0096	19	0.0139	16	0.0124	19
wWhiskers	0.0121	30	0.0147	30	0.0143	30
wHP	0.0127	28	0.0137	28	0.0159	28
wWHP	0.0149	23	0.0159	23	0.0182	23

All GD: all 35 geometric descriptors. RFE: geometric descriptors selected by the recursive feature elimination algorithm. Boruta–Shap: geometric descriptors selected by the Boruta algorithm based on Shapley values. wWhiskers: geometric descriptors resulting from the exclusion of those associated with the action unit Whiskers change. wHP: geometric descriptors resulting from the exclusion of those associated with the action unit Head position. wWHP: geometric descriptors resulting from the exclusion of those associated with the action units Whiskers change and Head position. N: number of geometric descriptors.

Regression models used total FGS scores (ratio 0–1.0). Data are reported as mean squared error (MSE). The ‘Mean’, ‘Maximum’ and ‘Minimum’ aggregation functions were used to aggregate the values assigned to the same image by multiple raters.

**Table 4:** Predictive performance of models used for ordinal classification based on Feline Grimace Scale (FGS) scores and each of the five action units of the FGS.

Action units	MSE (Mode)	MSE (Maximum)	MSE (Minimum)
Ear position	0.0806	0.0895	0.2158
Orbital tightening	0.1092	0.1491	0.1343
Muzzle tension	0.3134	0.4677	0.3431
Whiskers change	0.2258	0.4552	0.1774
Head position	0.1674	0.2089	0.1465

Ordinal classification models used the scores for each AU (0, 1 or 2). Data are reported as mean squared error (MSE). The ‘Mode’, ‘Maximum’ and ‘Minimum’ aggregation functions were used to aggregate the scores assigned to the same image by multiple raters.

## Discussion

From laboratory animals in biomedical research, pet medicine, farm animal production to people who cannot self-report pain (e.g. infants and individuals with dementia), there is an urgent need in our society for automated acute pain assessment systems with smartphone applicability that are independent of observer, species, training, sex, etc. In this study, we proposed a three-component independent system for prediction of facial landmark position, computation of geometric descriptors and the prediction of FGS scores. Three CNN models (ShuffleNetV2, EfficientNetB0 and MobileNetV3 architectures) including preprocessing based on face alignment and Laplacian edge detection filter presented the best predictive performance with reasonable prediction time and model size that could be suitable for a smartphone application to predict facial landmark positions (Fig. 1, Supplementary Fig S2). For the prediction of FGS scores using computation of geometric descriptors, binary classification models achieved high accuracy (≥ 95%) and discriminatory ability between painful and non-painful cats (Table 2, Supplementary Figs. S6–S7). We found that regression models for total FGS scores and ordinal classification models for each AU scores provided different MSE depending on the specific AU involved (Tables 3, 4). This system, using CNN and ensemble learning models applied explicitly to a validated grimace scale, showed promising results for automated pain assessment in cats with smartphone applicability as it can predict FGS scores with excellent accuracy and discriminatory ability, and minimal error (Table 2, Supplementary Figs. S6–S9). The technology can also now be further developed as a backbone model for grimace scales in other mammalian species.

The use of automated methods for recognition of pain using facial expressions and grimace scales is an exciting field of research. Indeed, there has been an interest in solving the problem (pain assessment) using binary classifications and there are several examples in the literature. Early work in rats included a partially automated approach, the Rodent Face Finder that generates picture files from videos for pain assessment^24^. A CNN model was able to categorize images of white mice using binary outcomes (no pain or pain) with high accuracy (94%)^19^. Another model in mice recognized the absence or presence of postsurgical pain with 99% accuracy^25^. Geometric landmarks were used successfully to quantify changes in facial shapes associated with pain in a small number of domestic shorthaired cats before and after ovariohysterectomy^26^ and/or administration of analgesics using the anatomy of the cat facial musculature and expressions of the cat’s Facial Action Coding System (FACS)^27^. The same model backbone was recently applied to classify painful versus non-painful cats using a landmark-based (using multi-region vectors and Multilayer Perceptron neural network based on manually annotated landmarks) or deep learning-black box (ResNet50 architecture using raw images without landmarks) approaches with and without data augmentation and face alignment. Both methods presented similar accuracy of above 72%^28^. Contrarily, our system was built explicitly on a robust semi-automatic annotated dataset and a well-designed two-phase approach using a validated acute pain scoring system in cats, the FGS^20^. Our data included total FGS and AU scores provided by image assessment when studies were performed. The dataset included different sources of naturally occurring pain in cats of different age, coat-color and type, breed, and sex with high accuracy (close to 96%). In our study, we did not evaluate any black-box models, which are less labor-intensive but do not provide background information for classification decisions. It is beyond the aim of our study to review the literature on facial recognition and pain assessment in animals but it is clear that there is a need for studies using automated methods of pain assessment using grimace scales that provide more information than binary classifications with for example, objective grimace scale scores using validated scales. Our present study provides prediction of landmark positions and actual FGS scores including for each AU (i.e. degrees of pain). Previous work in sheep using support vector machines resulted in accuracy of only 67% when using changes in facial expressions and nine AU for pain assessment^29^.

Recently, the development and validation of a two-component software platform that simplifies and standardizes mouse grimace analyses have been published using a large number of images^16^. It detects the mouse face/body (RetinaNet architecture) and predicts Mouse Grimace Scale (MGS) scores (ResNet50 architecture) using predictive performance as the outcome. In our study, model size and prediction time were also important for outcomes as these parameters are fundamental during smartphone integrations. For this reason, our proposed system leveraged fast and light CNN and ensemble learning models. The prediction of MGS scores was performed using a black-box method and it is not possible for the user to know how the CNN predicts MGS scores, especially in the case of unexpected scores. Our system explicitly involves the prediction of facial landmarks, calculation of geometric descriptors and FGS scores in CNN models based on semi-automatic annotations, geometric transformations and original FGS scores. Considering that the FGS provides a cut-off for the administration of analgesia, our system is able to alert the end-user when the cat is sufficiently in pain to require intervention with high discriminatory ability (AUROC of 97%). The combination of these factors allowed us to find highly accurate and discriminatory models using a relatively small dataset. Therefore, our system could identify and explain unexpected scores as geometric descriptors can be calculated and identified separately to predict FGS scores. Additionally, our system assumes that the user will always present a cat face in frontal position without a component of image pre-validation that recognizes the cat’s face itself. On the other hand, our work was more laborious than the MGS software as it required careful annotation of each image for the prediction of facial landmark positions. Manual annotations are time-consuming, but they may account for differences in facial morphology, breed and species differences.

In cats, pain has been historically neglected, under-recognized, under-diagnosed, and under-treated^30^. The knowledge of feline pain management has evolved with the advent of pain scoring systems. However, published behavior-based scales can be long and time-consuming and, in some cases, they are only valid for a single type of pain or have only undergone partial validation. Our proposed automated system using the FGS may overcome these limitations, especially with the potential of a user-friendly smartphone application that could widespread its use. Of interest, whiskers change and muzzle tension have consistently presented lower inter-rater reliability compared with the other AUs^8,17,20–22^. This information was corroborated for the prediction of AU scores with the largest prediction errors observed for muzzle tension and whiskers change. On the other hand, whiskers change and head position presented the largest prediction errors while orbital tightening and muzzle tension presented the lowest prediction errors (Fig. 1, Table 4). This finding might demonstrate possible inconsistencies with manual annotation of landmarks related to whiskers change and head position, which did not affect accuracy of pain scoring. The XGBoost binary classification models that did not include geometrical descriptors linked to whiskers change and head position still reached an accuracy of 94% and 93%, respectively (Table 2). Regression models without these two AUs presented MSE of 0.0121 and 0.0127 when using the mean of total FGS scores assigned to each image (Table 3).

The advantages of our system can be summarized into three main elements. First, the landmark-based approach using the FGS and its consequent geometric descriptors obtained models with good predictive performance using a small dataset and training times. This approach allows each AU to be scored separately, or even not scored at all, with minimal impact on accuracy. The results can be fine tuned after prediction of FGS scores from images as scores for each AU and the corresponding geometric descriptors are provided and could explain unexpected scores to the end-user (i.e. non-black-box approach). Second, each component/functionality is independent; therefore, prediction of facial landmark position, computation of geometric descriptors or prediction of FGS scores could be performed independently of one or another. Third, each component can be independently improved. For example, component 1 (prediction of facial landmark positions) was improved by including preprocessing based on face alignment and edge detection filter, and/or models’ structural transformations.

This study has limitations. As mentioned before, we did not use black-box models or a preliminary phase that includes facial recognition of a cat. It should be noted that predictive performance was best using frontal face positions. It is expected that predictive performance will be compromised with partial frontal or side position images. In other words, a smartphone application would require some guidance to end-users with face alignment of the cat. We computed geometric descriptors and included transformations that may account for geometric variations and face morphology due to age (adult cats versus kittens), sex, coat color, breed, etc. to minimize these effects on predictive performance while reducing variations in image brightness, contrast and/or color balance. For the prediction of facial landmark positions, the ShuffleNetV2, EfficientNetB0 and MobileNetV3 architectures using face alignment showed the best predictive performances. However, this type of preprocessing has the disadvantage that it requires semi-automatic pre-annotation of 10 of the 37 landmarks, or prior training of models for the prediction of these 10 landmarks. The number of landmarks could be reduced to only 2, if figures containing only the face or the relevant features for the determination of the FGS AU were to be used. The development of a smartphone application with automated acute pain detection capabilities should incorporate all three components presented in this study. Ideally, the predictive performance should not be reduced by integration of these components for this purpose. If the latter is the case, models for prediction of FGS scores based on facial subregions or the use of a heatmap-based CNN architecture for detecting facial landmarks could be a potential solution for this issue. Additionally, the high accuracy of our models for the prediction of FGS scores were only possible with the use of real-time or image pain scores by raters who were veterinarians with experience in feline acute pain assessment. Finally, we hope that this model backbone could be applied to other mammalian species. However, changes in facial morphology, geometric variations, dataset size and heterogeneity, species, and the use of other validated grimace scales may affect study outcomes even when applying a similar methodology.

In conclusion, for the prediction of facial landmark positions, models using ShuffleNetV2, EfficientNetB0 and MobileNetV3 architectures showed the best predictive performances. Image preprocessing with face alignment and Laplacian edge detection filter improved predictive performance when compared to preprocessing without face alignment or based on other filters, respectively. For the prediction of total FGS scores and each AU, XGBoost models using binary classification and 35 geometric descriptors showed the best predictive performance with high accuracy (95.5%). Principal Component Analysis showed a well-defined distinction between painful and non-painful cats. In summary, deep-learning-based models for facial landmark prediction and ensemble learning models for FGS score prediction presented suitable sizes and prediction times, and excellent predictive performance and accuracy to discriminate painful and non-painful cats. This technology can be used for subsequent development of a smartphone application for automated acute pain assessment in cats based on the FGS.

## Methods

**Figure 2:**
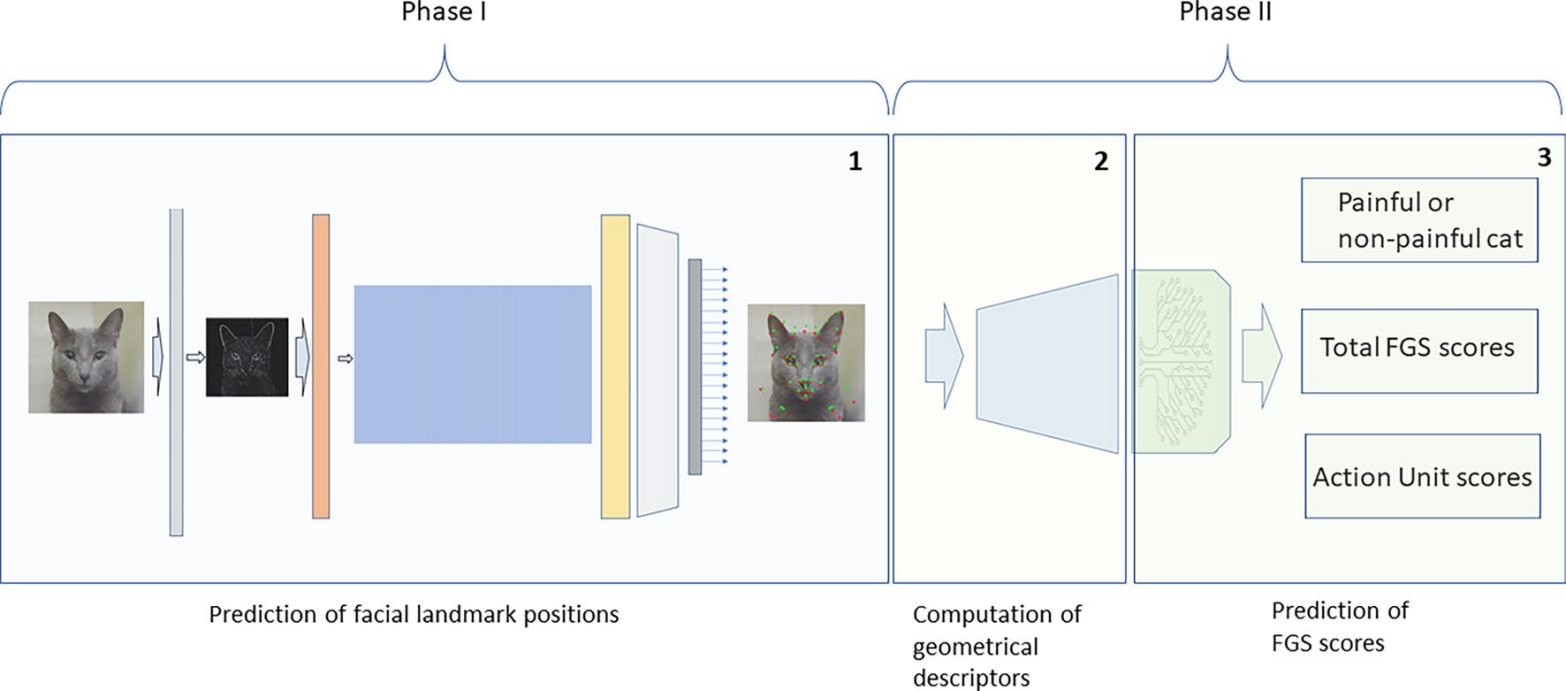
Schematic diagram of the steps and procedures for automated prediction of facial landmarks and Feline Grimace Scale (FGS) scores using facial images of domestic cats with and without naturally-occurring pain. Phase I involved component 1 (prediction of facial landmark positions). Phase II involved component 2 (computation of geometrical descriptors) and component 3 (prediction of FGS scores). In component 1, the gray and orange bars represent preprocessing with edge detection filters and face alignment, respectively; the blue rectangle, yellow and light grey bars represent convolutional neural networks for prediction of 37 facial landmarks. In component 3, the green rectangle represents the XGBoost models.

This study was divided in Phases I and II. Phase I involved the prediction of facial landmarks position and Phase II, the prediction of FGS scores (Fig. 2).

### Datasets

In Phase I, a dataset including 3447 facial images of cats from three main sources (research studies, FGS mobile phone application and Kaggle dataset) was used. Images from research studies (n = 1655) were collected from cats with or without different degrees of naturally-occurring pain during six clinical trials. These studies are named herein as A^20^, B^17,31^, C^21^, D^32^, E^33^ and F^34^; they were performed after review and approval by the institutional animal care and use committee of the Faculty of Veterinary Medicine, Université de Montréal (17-Rech-1863, 18-Rech-1825, 17-Rech-1890, 20-Rech-2068, 20-Rech-2075 and 21-Rech-2132, respectively). Dataset included cats of different coat color, age, sex and breed. Still images had been collected from video recordings of cats while they were undisturbed in their hospital cages at different time points (i.e. before and after surgery; before and after administration of analgesia). Images were also collected from the pool of images voluntarily submitted by users of the FGS mobile phone application (n = 1092) and from the open-access Kaggle dataset (n = 700) (www.kaggle.com/crawford/cat-dataset). Data were available in “.png” format.

In Phase II, a dataset including images from the research studies dataset and their respective FGS scores (n = 1188 out of 1655 images) was used. Scores were given by one or more raters during data collection of studies A–F^17,20,31–33^. Data were organized into an Excel file containing the image and rater identification as well as their scores for each AU of that image. Raters were veterinarians experienced with acute pain assessment in cats and the use of the FGS (6 females and 2 males). Action units were scored as ‘0’, ‘1’or ‘2’, where ‘0’ = AU is absent; ‘1’ = moderate presence of AU or uncertainty over its presence or absence; and ‘2’ = obvious presence of AU^17,20,31–33^. Total FGS scores were calculated as the sum of all AUs divided by the maximum possible score based on the number of AUs that were scored for each image; thus, total FGS scores were available as ratios.

### Landmark positions and semi-automatic annotations

**Figure 3:**
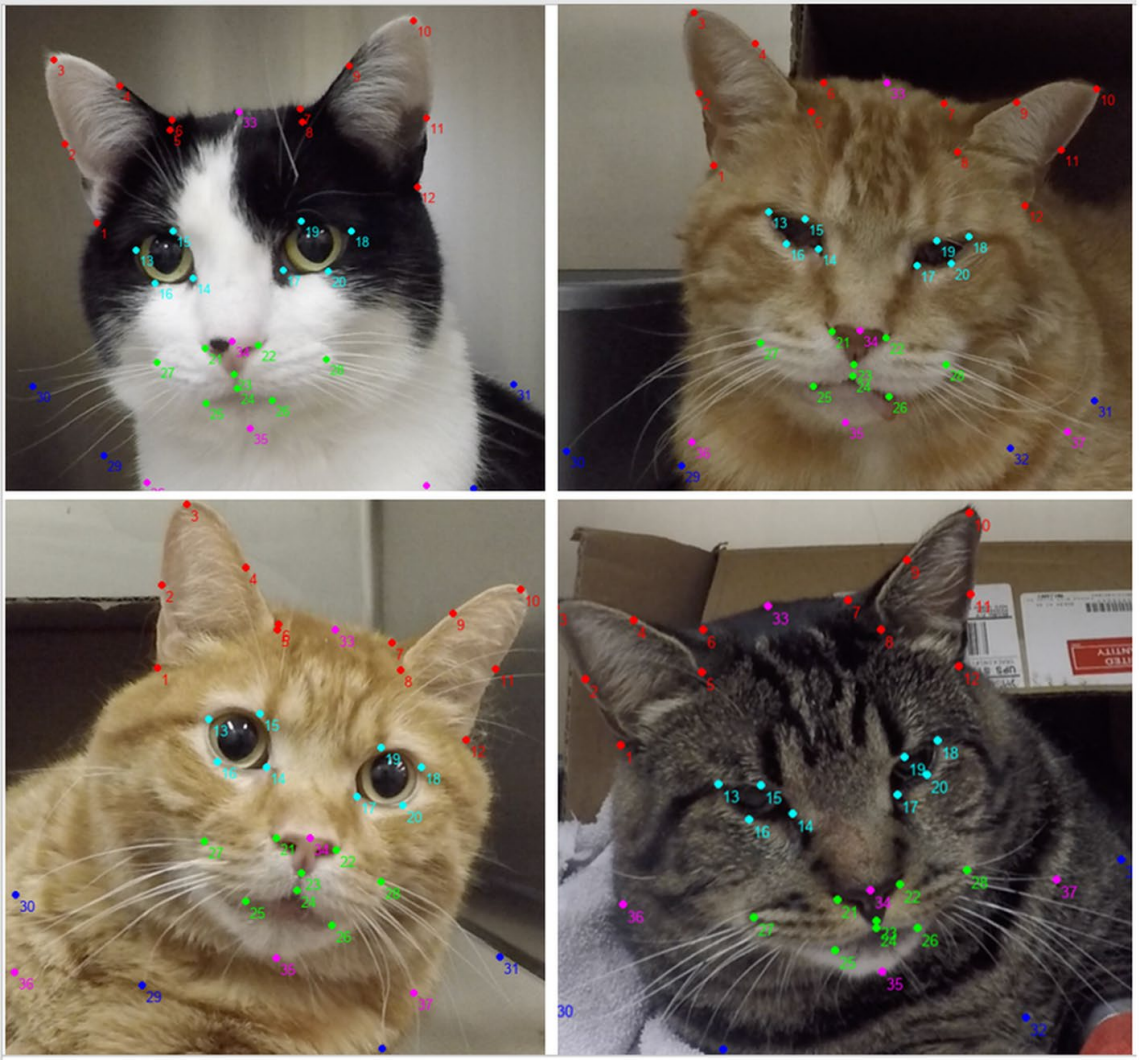
Examples of images of cats with 37 facial landmarks based on the five action units (AU) of the Feline Grimace Scale (FGS). Each AU is represented by a different color. Ear position: red. Orbital tightening: light blue. Muzzle tension: green. Whiskers change: dark blue. Head position: pink. Note the different facial expressions between non-painful (images on the left) and painful (images on the right) cats. Painful cats generally present with lowered ears rotating outwardly, squinted eyes, tense muzzle and whiskers, and lowered head position in relation to the shoulders.

A total of 37 facial landmarks were defined based on the five AU of the FGS by two investigators (BM and PVS) (Fig. 3). Landmarks (annotation points) were added to facial images of cats to visually delineate each AU while observing the changes in these landmarks’ positions from images of non-painful and painful cats. Once landmarks were defined, they were numbered and their anatomical location described (Supplementary Figs. S10–S11; Table S1). Thereafter, a software was specifically designed by one of the investigators (MM) for semi-automatic annotation of the 37 facial landmarks on each image. Data from each landmark (coordinate x and y) were saved automatically in .txt format and converted to .xlsx format using one file converter.

The reliability of these annotations using this software was confirmed. Briefly, three raters annotated 20 random images twice four days apart. Inter- and intra-rater reliability were calculated using weighted Kappa and intraclass correlation coefficient, respectively, showing good reliability of the tool (unpublished data). Subsequently, all 3447 images were annotated by one of the investigators who participated in the reliability trial (SM) using such software. On average, 50 images were annotated daily for 69 days.

### Phase I—Prediction of facial landmark positions using convolutional neural network models

Convolutional neural network models (CNN) were developed by two investigators (MS and MM) to predict the coordinates of the 37 facial landmark positions^23,35^. A total of 3447 facial images were used in this phase.

#### Dataset augmentation and transformations

**Figure 4:**
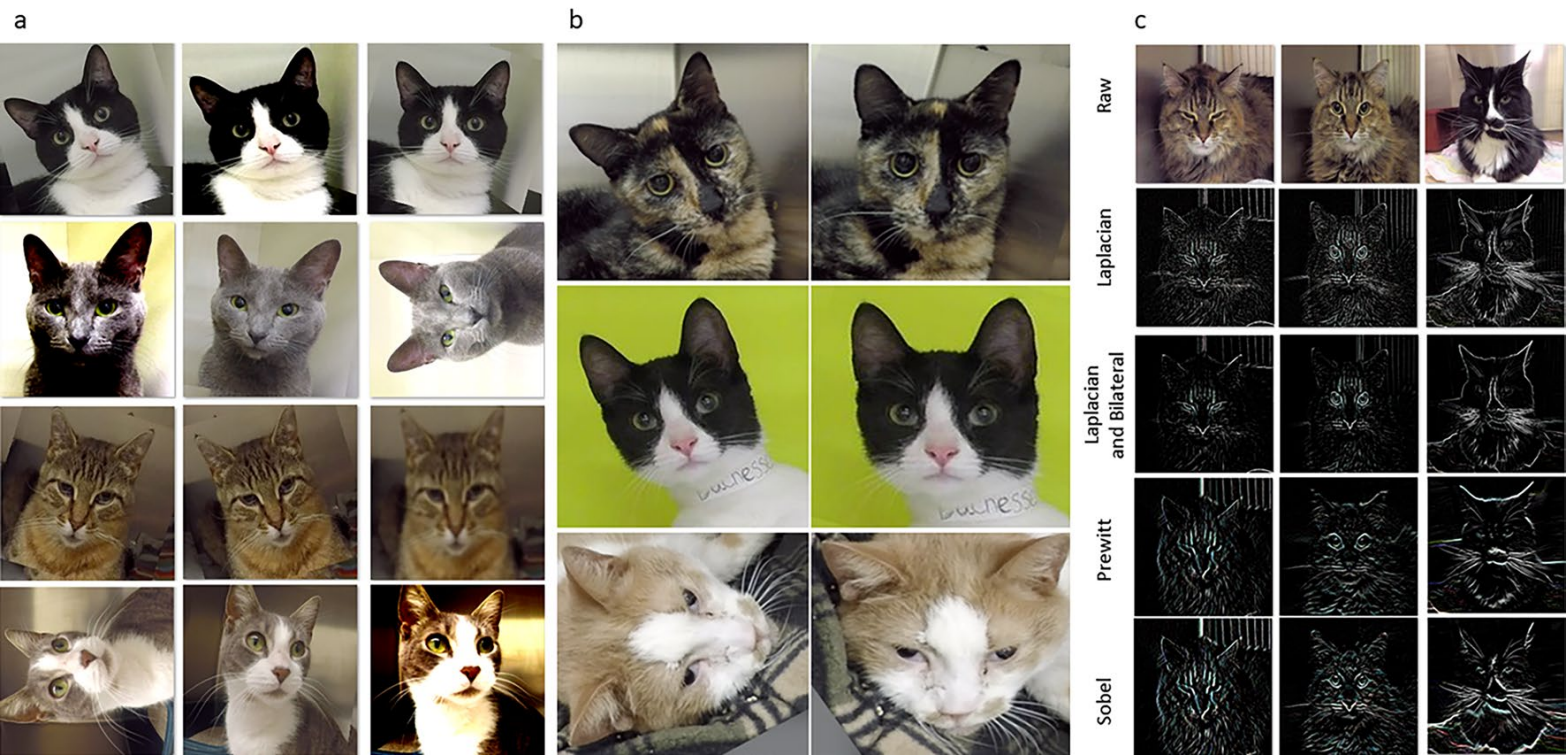
Examples of facial images of cats after dataset augmentation and transformations. (**a**) Images generated after randomly introducing geometric and color-space transformation (including Gaussian blur filter) to the original images for dataset augmentation. (**b**) Images before (left) and after (right) face alignment. The 2D face alignment was performed before the application of convolutional neural network models for facial landmark detection. Two separate groups of landmarks were used: landmarks 13, 14, 17 and 18 were used for calculation of the rotation angles; landmarks 3, 10, 29, 30, 31, 32, 36 and 37 were used for cropping and resizing so faces have approximately the same orientation and size. The presence of all facial landmarks was considered more important than the size or the position of the face. (**c**) Images before and after edge detection filters. Images were preprocessed independently by each of four edge detection filters before application of convolutional neural network models for facial landmark detection. Edge detection filters were used to reduce the influence of variations in brightness, contrast or color balance on the coordinate prediction. From top to bottom: raw images and images preprocessed by Laplacian filter, Laplacian and Bilateral filters, Prewitt filters and Sobel filters, respectively. Kernels of 3 × 3 size were used for Sobel, Prewitt and Laplacian filters. The latter was used directly or after application of the Bilateral filter to reduce noise. The parameter values for Bilateral filter were 3 for the pixel neighborhood diameter, and 100 for SigmaColor and SigmaSpace. For the other filters, the weighted sum of the derived images was calculated after convolving the image with its vertical and horizontal masks.

The dataset was augmented for increased size and heterogeneity and consequent suitability for deep learning. Geometric and color-space transformations were randomly introduced to the original images (Fig. 4)^36^. Geometric transformations included: rotation, flips, shearing, and face cropping and resizing. Color-space transformations included: contrast, sharpness, brightness and color balance. Gaussian blur filter was also applied. The values of the parameters linked to these transformations were randomly applied in the following ranges using predefined probability distributions: rotation [(3–19), (341–357)]; shearing (− 0.16, 0.18); flips (left–right flip, rotation − 90, rotation − 270); contrast (0.6–2); sharpness (0.4–8); brightness (0.7–1.6); color balance (0.2–3.5) and Gaussian blur (1.05–2.9)^36,37^. As part of geometric transformations, face cropping and resizing predicted the boundaries of faces using the Haar Cascade method implemented in OpenCV^37,38^. Each image was cropped using a scale factor of 1.01 and minNeighbors parameter of 5. Only cropped images having an area A greater than 0.4*Ao, where Ao is the area of the original image, were accepted. Accepted images were then resized back to their original size. Two main image preprocessing transformations were used including face alignment and edge detection filters (Fig. 4).

#### Models design

Following exploration of multiple CNN-based models with different architectures and trained on different augmented datasets, four Keras models pretrained on ImageNet (NASNetMobile, EfficientNetB0, MobileNetV2 and MobileNetV3) and other CNN-based models including ShuffleNetV2 were used. Several changes were introduced in these models after removing the last layer. The blocks of the NASNet architecture (NASNetMobile) were designed by the authors using a method based on the Neural Architecture Search (NAS)^39^. These blocks were stacked to form a high dimensional architecture, which was trained and evaluated on another image classification dataset, ImageNet. Features learned by NASNet improved object detection^40^. EfficientNetB0 was designed using a multi-objective neural architecture search that optimizes both accuracy and floating-point operations using inverted bottleneck residual blocks and squeeze-and-excitation blocks^41^. MobileNetV2 and MobileNetV3 were used with different adaptations for landmark prediction as in previous studies^42,43^. MobileNetV2 was designed using bottleneck and inverted residual blocks containing pointwise and depthwise convolutions to contribute to image classification, object detection and semantic segmentation^44^. For MobileNetV3, a platform-aware Neural Architecture for block-wise search and the NetAdapt algorithm were used to optimize the number of filters per layer^45^. Non-pretrained ShuffleNetV2 models were designed using pointwise group and depthwise convolutions, bottleneck-like structures, and a channel shuffle operation^46^.

Initially, most models were built with a simple structure at the top of the adopted CNN architecture consisting of two layers, the GlobalAveragePooling2D (GAP2D) and the dense output layers. Then, three types of transformations were added: (1) one or two dense layers below the output layer; (2) the GAP2D layer was replaced by a flatten layer; and (3) a block of parallel convolutional layers was inserted between the GAP2D or flatten layer and the previous layer. These parallel convolutional layer blocks had symmetric, asymmetric or hybrid (symmetric and asymmetric) kernels. The activation function ReLu was used with the padding ‘same’.

#### Training and evaluation of the models

The best models were chosen after a first training session in which multiple values of 2 groups of structural hyperparameters were tested, some related to the new layers added (e.g. number of neurons, layers) and others directly related to the CNN architectures (e.g. width multiplier, bottleneck ratio). Then, to increase the performance of these models, those with the best values of the hyperparameters related to the training setup such as validation loss, learning rate, batch size, optimizer, callback hyperparameters, among others, were selected.

Three metrics were used to evaluate the models that could be suitable for a smartphone application on a test set of 120 randomly selected images:

- Model size referred to the number of parameters or storage space required by the model.
- Prediction time referred to the time taken to predict the positions of all facial landmarks in an image (inference time plus preprocessing time).
- Predictive performance was calculated using the Normalized Root Mean Square Error (NRMSE). The NRMSE (%) is defined as the average normalized Euclidean distance between the predicted landmarks and the corresponding ground truth landmarks (i.e. by semi-automatic annotation) expressed as percentage. A lower NRMSE value in this context indicates a better fit between the predicted and ground truth landmarks.

Equation for the calculation of NRMSE (%):

$$NRMSE(\%)= NRMSE\times 100$$

$$NRMSE(\%)=\left(\frac{1}{N}{\sum }_{k=1}^{I}\sum_{i=1}^{L }\frac{\sqrt{{\left({x}_{i,k}^{p}-{x}_{i,k}^{g}\right)}^{2}+{\left({y}_{i,k}^{p}-{y}_{i,k}^{g}\right)}^{2}}}{{d}_{n,k}}\right)\times 100$$

$${d}_{n,k}=\sqrt{{\left({x}_{l33,k}^{g}-{x}_{l34,k}^{g}\right)}^{2}+{\left({y}_{l33,k}^{g}-{y}_{l34,k}^{g}\right)}^{2}}$$

$N$: Total number of landmarks in the test dataset, $N=IL$

$I$: Total number of images in the test dataset.

$L$: Number of landmarks per image (37).

$\left({x}_{i,k}^{p}, {y}_{i,k}^{p}\right)$: Coordinates of the predicted position for landmark *i* in image k.

$\left({x}_{i,k}^{g}, {y}_{i,k}^{g}\right)$: Coordinates of the “ground truth” position for landmark *i* in image k.

${d}_{n,k}$: Normalization distance between the landmarks 33 and 34 in image k.

$\left({x}_{l33,k}^{g}, {y}_{l33,k}^{g}\right)$: Coordinates of the “ground truth” position for landmark *l33* in image k.

$\left({x}_{l34,k}^{g}, {y}_{l34,k}^{g}\right)$: Coordinates of the “ground truth” position for landmark *l34* in image k.

#### Phase II—Prediction of FGS scores based on geometric descriptors and ensemble learning models

XGBoost models were implemented as part of the ensemble learning strategy by one investigator (MS) to predict FGS scores based on geometrical descriptors calculated from the facial landmarks strategy. A total of 1188 images and their respective FGS scores were used.

#### Geometric descriptors

**Figure 5:**
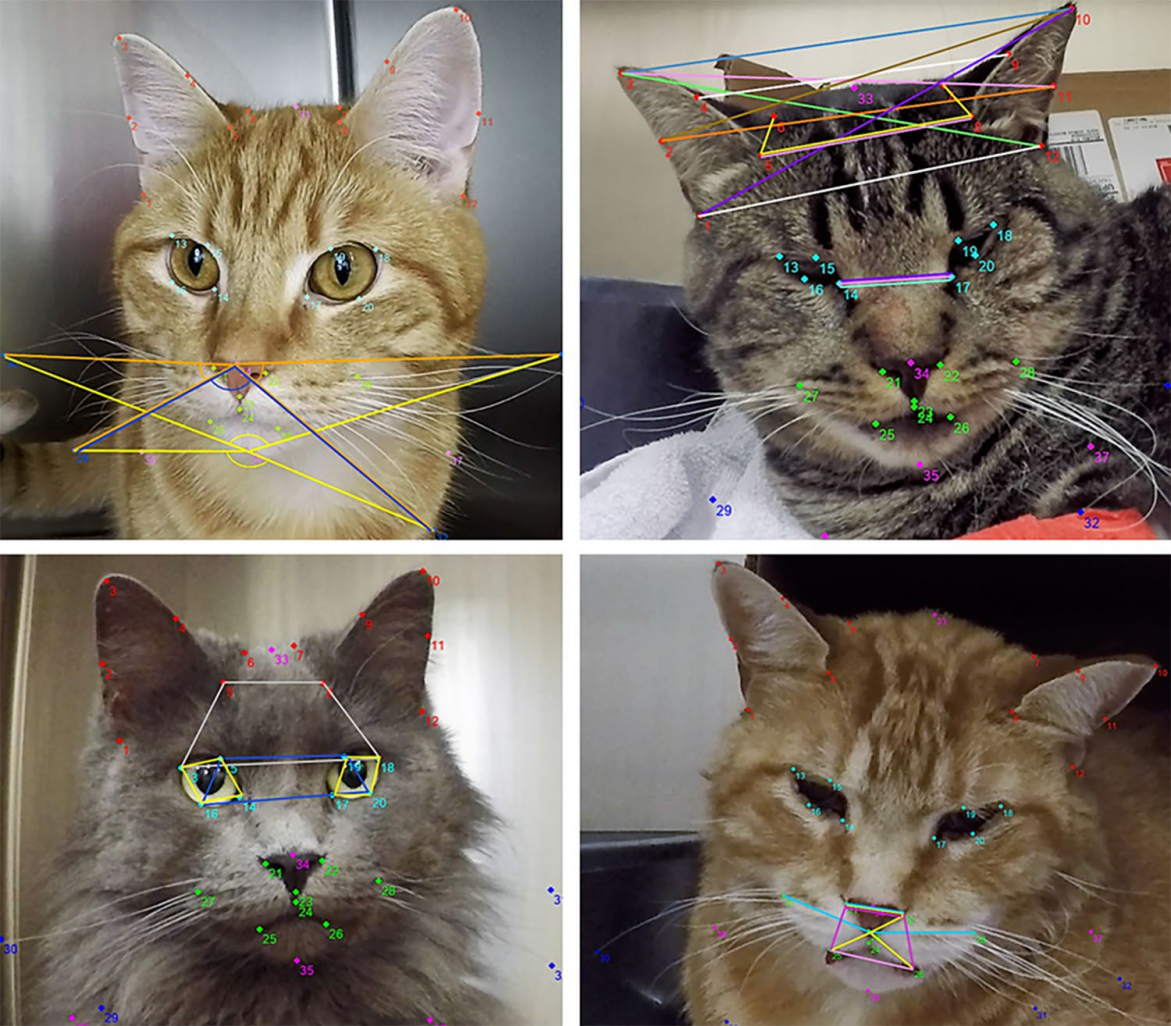
Examples of geometric descriptors calculated from 37 facial landmarks based on the five action unites (AU) of the Feline Grimace Scale. Each AU is represented by a different color. Ear position: red. Orbital tightening: light blue. Muzzle tension: green. Whiskers changes: dark blue. Head position: pink. Top left: lines between landmarks used to calculate distance ratios for AU whiskers change. Top right: lines between landmarks used to calculate distance ratios for AU ear position. Bottom left: lines between facial landmarks used to calculate angles for AU orbital tightening. Bottom right: lines between landmarks used to calculate distance ratios for AU muzzle tension. Description of each facial landmark and additional examples of geometric descriptors are available in Supplementary Table 1 and Figs. S10–S16.

Three types of geometric descriptors were defined: angles, ratios of distances (between landmarks) and ratios of areas (quadrilaterals whose vertices were landmarks) (Fig. 5, Supplementary Figs. S12–S16). Most geometrical descriptors were averages of geometric properties of the same type (angles, ratios of distances or areas) but calculated from different sets of facial landmarks. Thus, the geometric morphology of the cat’s face and its changes according to the pain severity was explored using 35 geometric descriptors that were later used for predicting FGS scores. This included a total of 10, 5, 8, 5 and 7 geometrical descriptors for ear position, orbital tightening, muzzle tension, whiskers change and head position, respectively.

#### XGBoost models

Three types of XGBoost models were implemented for the prediction of FGS scores using geometric descriptors:

- Binary classification models using ‘painful’ or ‘non-painful’ categories according to the total FGS score and cut-off for administration of analgesia (FGS scores ≥ 0.4/1 were categorized as ‘painful’)^20^.
- Regression models using total FGS scores (ratio; 0–1.0).
- Ordinal classification models using the scores for each AU (0, 1 or 2).

Each image had different scores that had been assigned by different raters. Therefore, aggregation functions were used to reduce the number of scores to a single score per image (‘AND’ and ‘OR’ rules for binary classification models; ‘Mean’, ‘Maximum’ and ‘Minimum’ rules for regression models; and ‘Mode’, ‘Maximum’ and ‘Minimum’ rules for ordinal classification models). The class imbalance observed with the application of ‘AND’ and ‘OR’ aggregation functions was considered for the determination of hyperparameter values "scale_pos_weight". The ‘Mode’ function was used to find the most common FGS score assigned to each action unit by different raters. Six combinations of geometric descriptors were evaluated: those containing all 35 geometric descriptors (All GD); those selected by the Recursive Feature Elimination (RFE) algorithm or Boruta algorithm based on Shapley values (Boruta-Shap); and those resulting from the exclusion of geometric descriptors associated with the AU whiskers change (wWhiskers), head position (wHP) or both (wWHP). The exclusion of geometric descriptors associated with whiskers change and head position was evaluated based on results of Phase 1 (see below). All models included geometric descriptors as independent variables.

Three general procedures were used to prevent overfitting in the final XGBoost models: hyperparameter tuning and cross-validation, hyperparameter alpha L1 regularization on weights and algorithms for feature subset selection. A total of five hyperparameters were used: number of trees, learning rate, L1 regularization parameter, maximum depth of trees and subsampling ratio for the training dataset and for the columns. Tuning was done using Grid-search^47^.

#### Training and evaluation of the models

Training and selection of the best values for the hyperparameters were performed for all models using Grid-search and cross-validation with shuffling and n_splits = 5. Recursive feature elimination is a greedy algorithm for the backward selection of predictors that was used along with the cross-validation procedure with shuffle and n_splits = 4 to select a subset of features that contributed the most to the performance of the model. In addition, Shapley values and the Boruta algorithm were used to select relevant features. Shapley values allowed the calculation of the average marginal contribution of each feature to the model predictions. The Boruta algorithm was based on randomized copies (shadow features) and the z-scores for Shapley values of each variable. Features that were significantly higher than this maximum z-score were considered relevant.

Principal component analysis (PCA) was performed for binary classification models to visualize the relationship of the covariance structure of 35 geometric descriptors to discriminate ‘painful’ and ‘non-painful’ cats.

Three metrics of predictive performance were used to select the best models on a test set of 100 randomly selected images:

- Accuracy and area under the receiver operating characteristic curve (AUROC) for the binary classification models
- Mean squared error (MSE) for the regression and ordinal classification models.

#### Softwares

Keras and Tensorflow were used as backend for deep learning methods^48,49^. XGBoost library was used for the gradient boosting machines^47^. OpenCV and PILLOW were used for digital image processing^37^. The other machine learning tasks were carried out with Scikit-learn^50^. BorutaShap package was used for Boruta feature selection method based on Shapley values^51^.

### Supplementary Information

Supplementary Information.

## Data Availability

The datasets generated and/or analysed during the current study are not publicly available due to the undergoing development of the mobile phone application, but are available from the corresponding author on reasonable request.
