# Supplementary material for: Fully automated deep learning models with smartphone applicability for prediction of pain using the Feline Grimace Scale
Source: Sci Rep. 2023 Dec 7;13:21584. doi: 10.1038/s41598-023-49031-2 (PMC10703818; doi:10.1038/s41598-023-49031-2)
Supplement: Supplementary file 1 — Supplementary Information. [file 41598_2023_49031_MOESM1_ESM.docx]

**Supplementary information**

**Fully automated deep learning models with smartphone applicability for prediction of pain using the Feline Grimace Scale**

*Steagall PV,^1,2*^ Monteiro BP,^1^ Marangoni S,^1^ Moussa M,^3^ Sautié M ^3^*

^1^Department of Clinical Sciences, Faculty of Veterinary Medicine, Université de Montréal, Saint-Hyacinthe, QC, Canada; ^2^Department of Veterinary Clinical Sciences and Centre for Animal Health and Welfare, Jockey Club College of Veterinary Medicine and Life Sciences, City University of Hong Kong, Hong Kong, China; ^3^Plateforme IA-Agrosanté, Université de Montréal, Saint-Hyacinthe, QC, Canada.

*Corresponding author: [pmortens@cityu.edu.hk](mailto:pmortens@cityu.edu.hk)

**Supplementary information - Results**

| 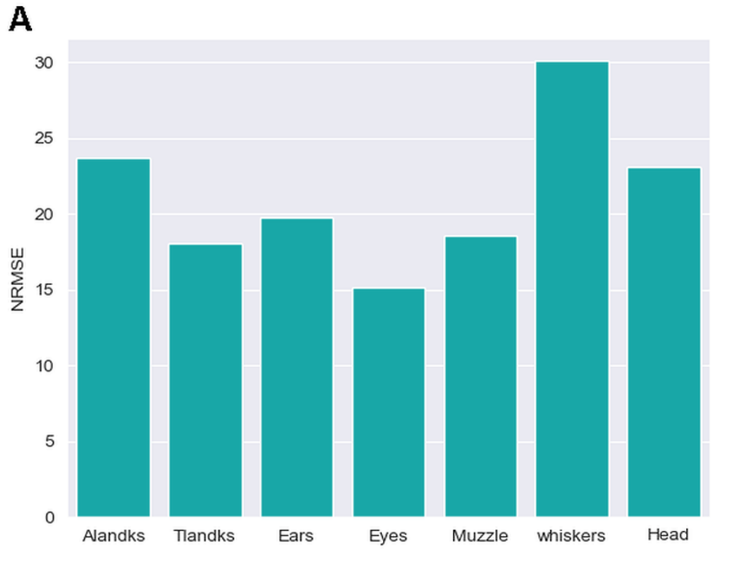 | 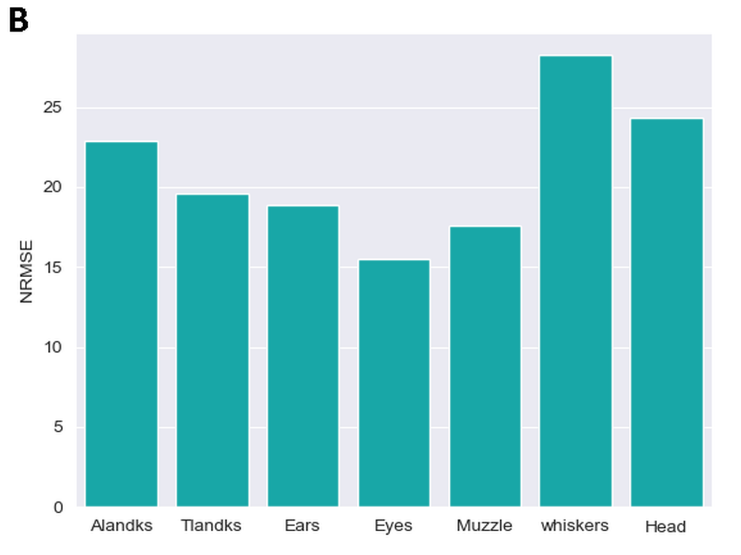 |
| --- | --- |
| 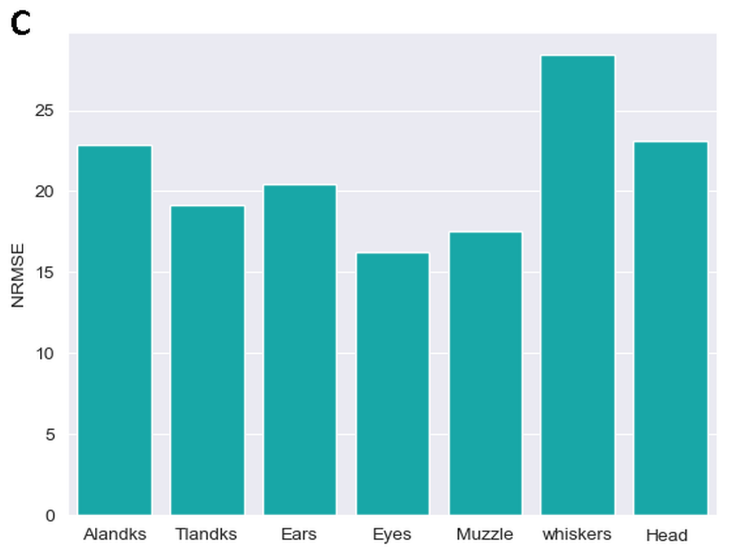 | 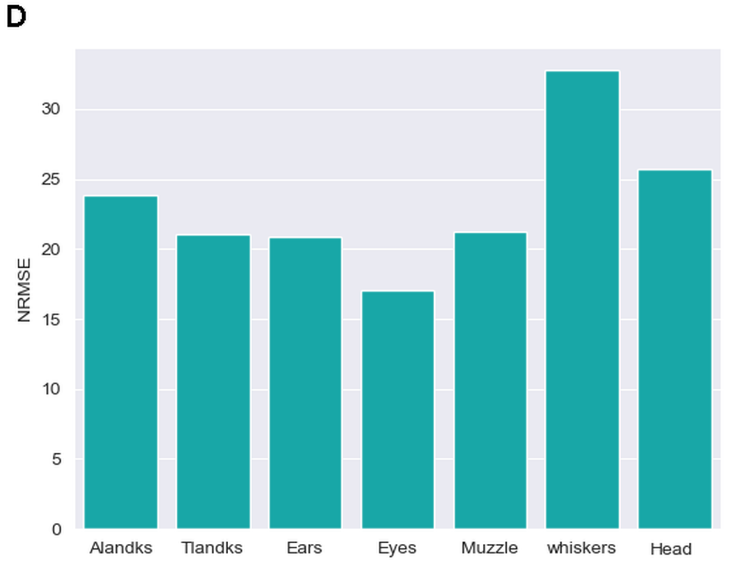 |
| **Figure S1.** Predictive performance (NRMSE (%)) for the landmarks linked to each of the five action units of the Feline Grimace Scale (Ear position, Orbital tightening, Muzzle tension, Whiskers change and Head position), as well as, to the 2 landmark subsets, Alandks: Alignment landmarks, Tlandks: Landmarks not used for face alignment. A: EfficientNetB0 model trained on dataset augmented by random color-space and geometric modifications. B: Minimalist version of MobileNetV3-Large model with image preprocessing by Laplacian filters. C: MobileNetV3-Large model with image preprocessing by Laplacian filters. D: ShuffleNetV2_0.5_1 model with a convolutional block of parallel layers and trained on a dataset augmented by random color-space transformations | |

| 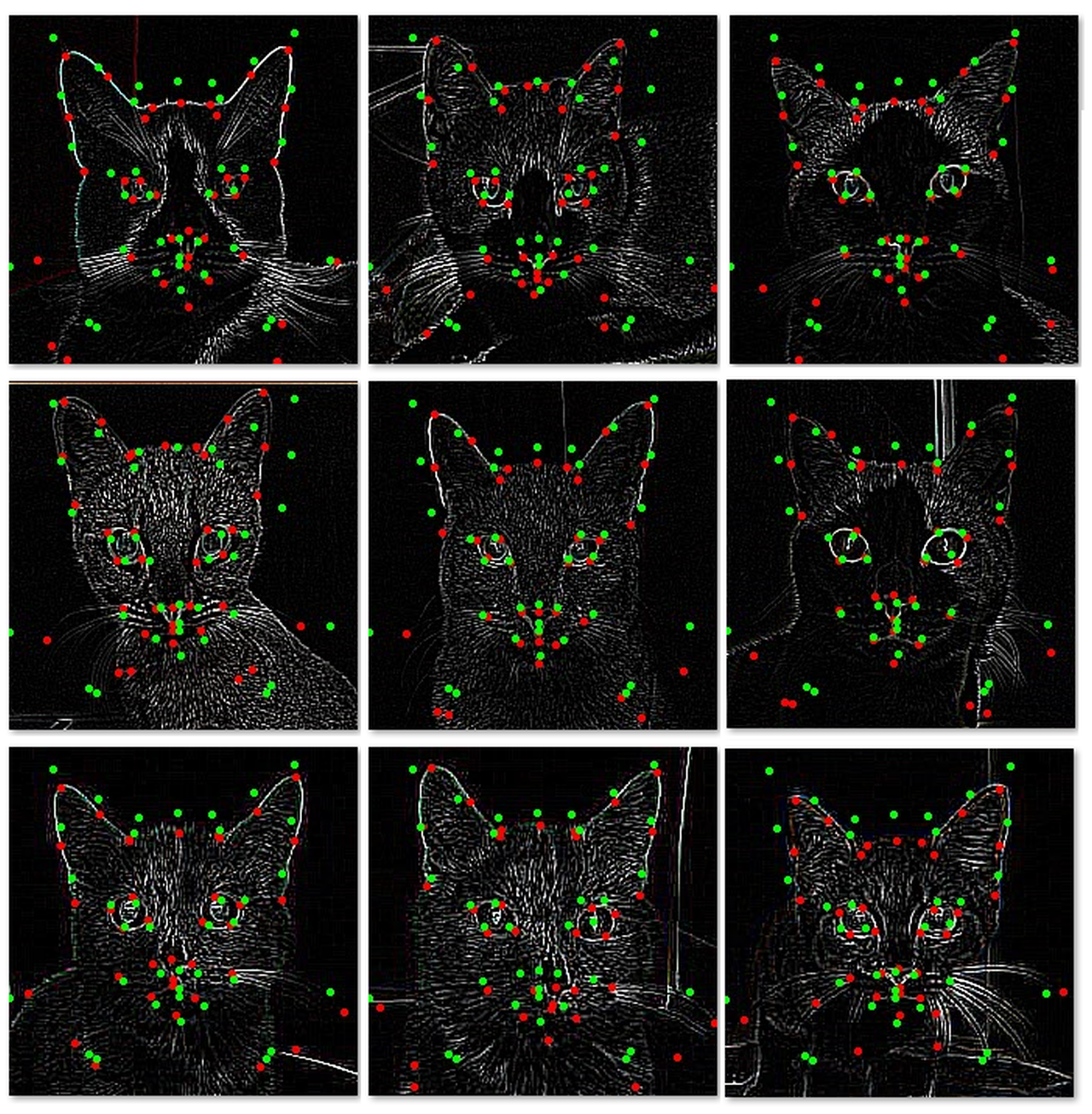 |
| --- |
| **Figure S2.** Green points: Predictions of a ShuffleNetV2_0.5_1 model on images preprocessed by Laplacian filter (size 3x3). For illustrative purposes the predictions were placed on the pre-processed images and not on the raw images. Red points: Ground Truth. |

| 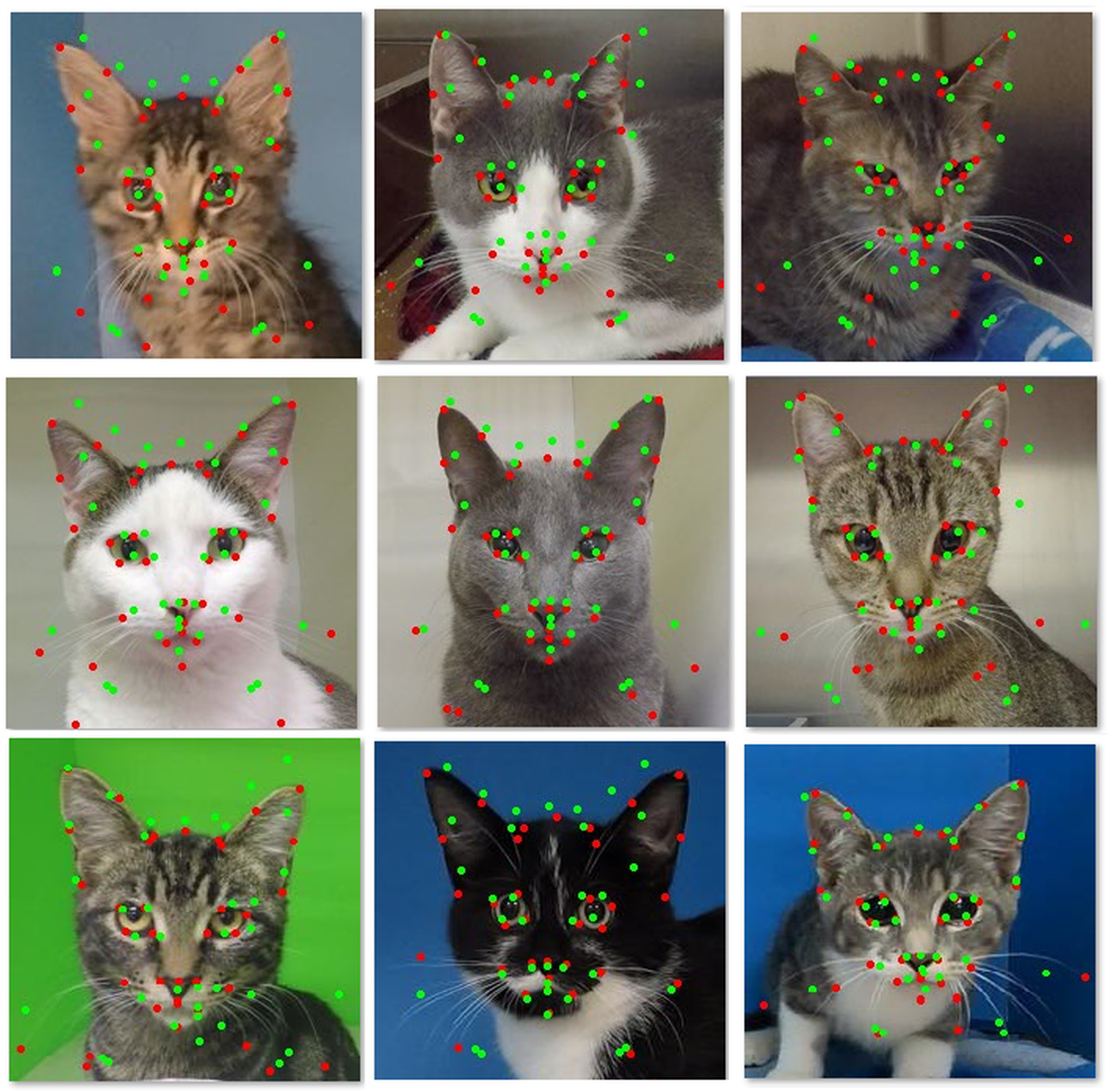 |
| --- |
| **Figure S3**. Green points: Predictions of EfficientNetB0 derived from the Keras pre-trained model of the same name. The 80 top layers of this model were trained on a dataset augmented by random color-space and geometric transformations. Red points: Ground truth. |

| 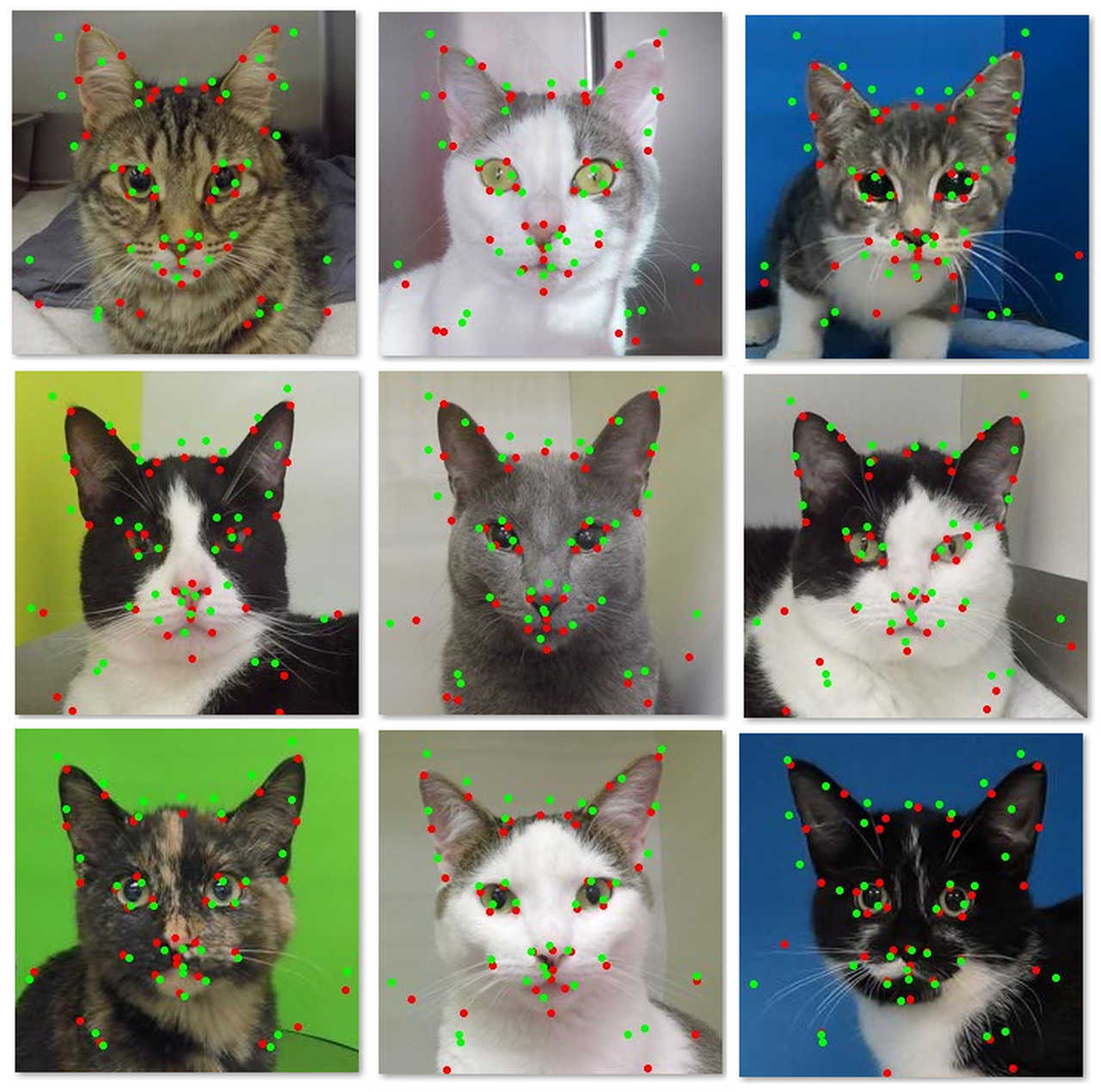 |
| --- |
| **Figure S4.** Green points: Predictions of MobileNetV3Large from the Keras pre-trained model of the same name. The 120 top layers of this model were trained on a dataset preprocessed by Laplacian filters. Ground truth: Red points. |

| 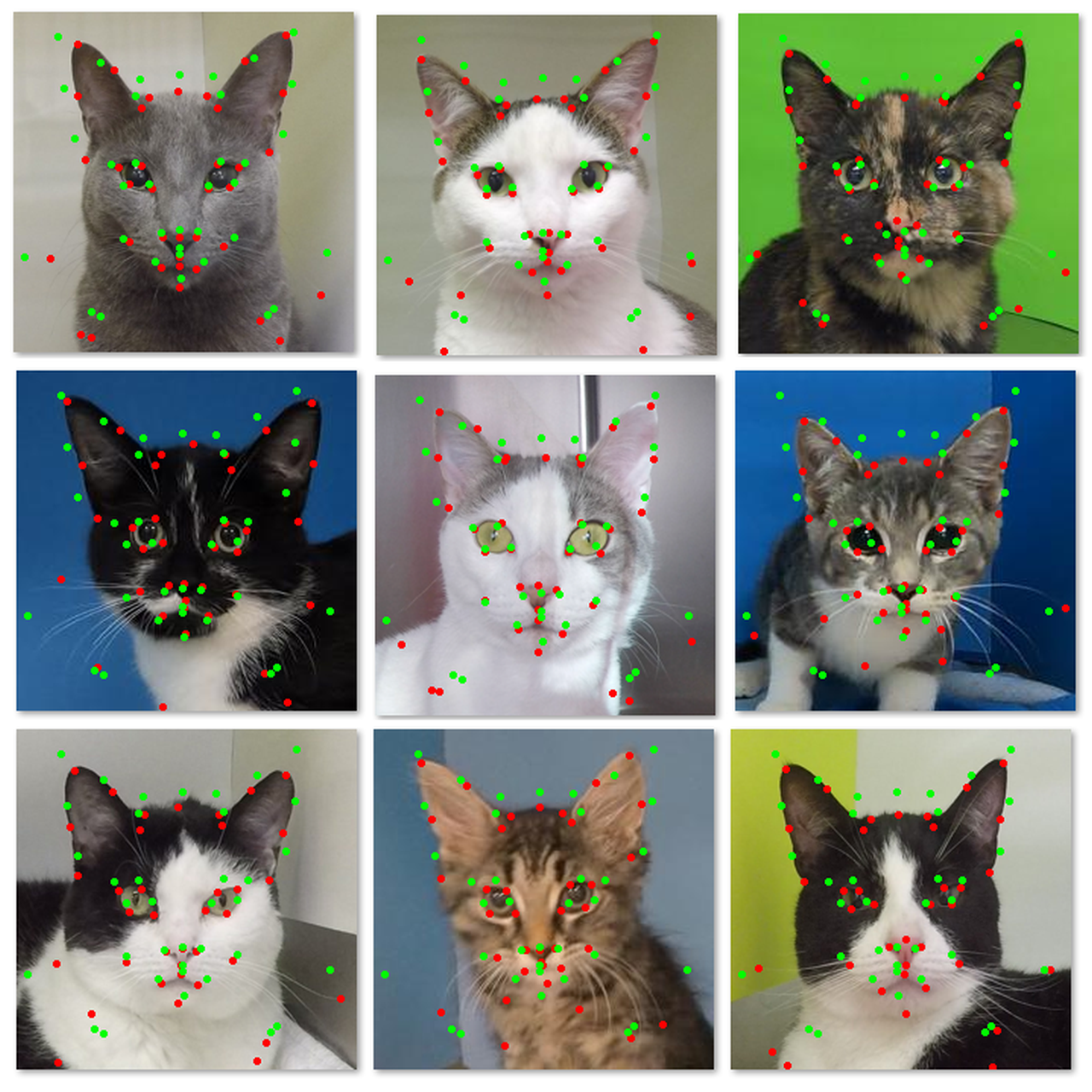 |
| --- |
| **Figure S5.** Green points: Predictions of the ShuffleNetV2_0.5_1 model. This model was trained on a dataset augmented by random color-space and geometric transformations. Red points: Ground Truth. |

| 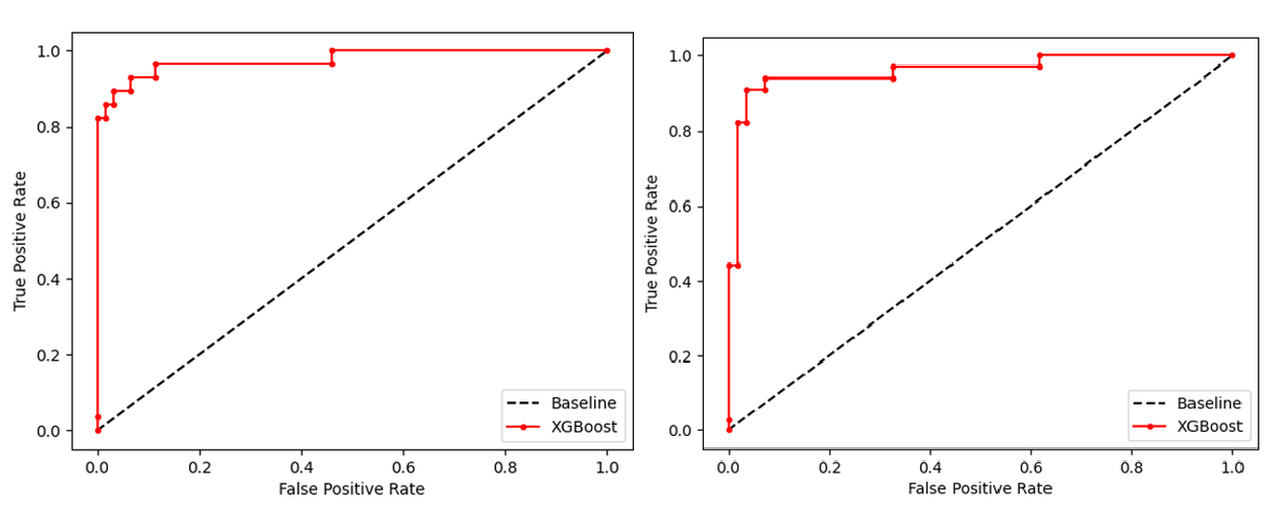 |
| --- |
| 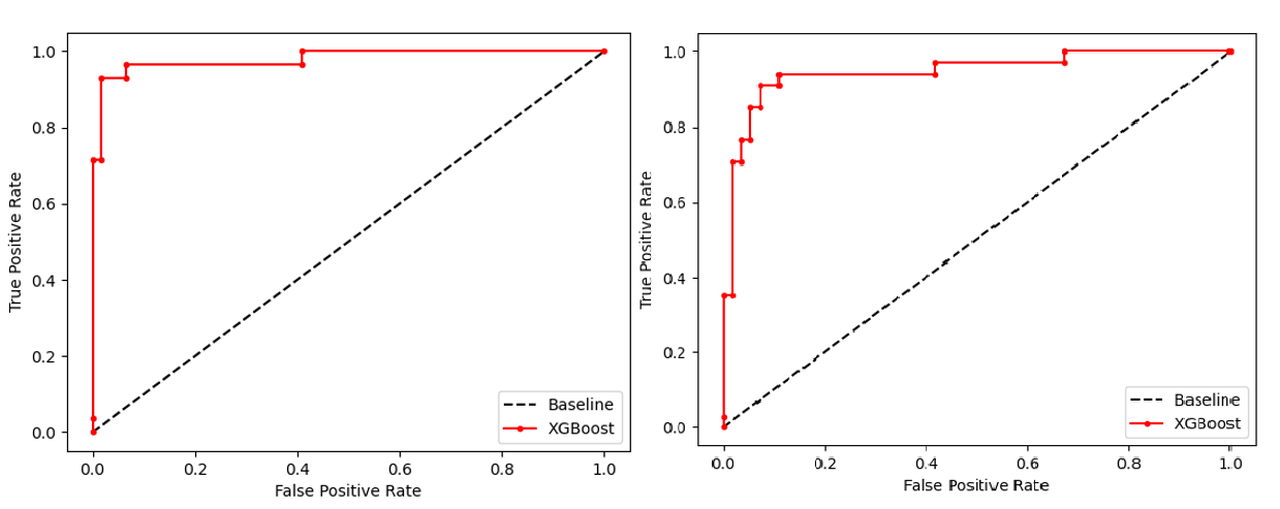 |
| 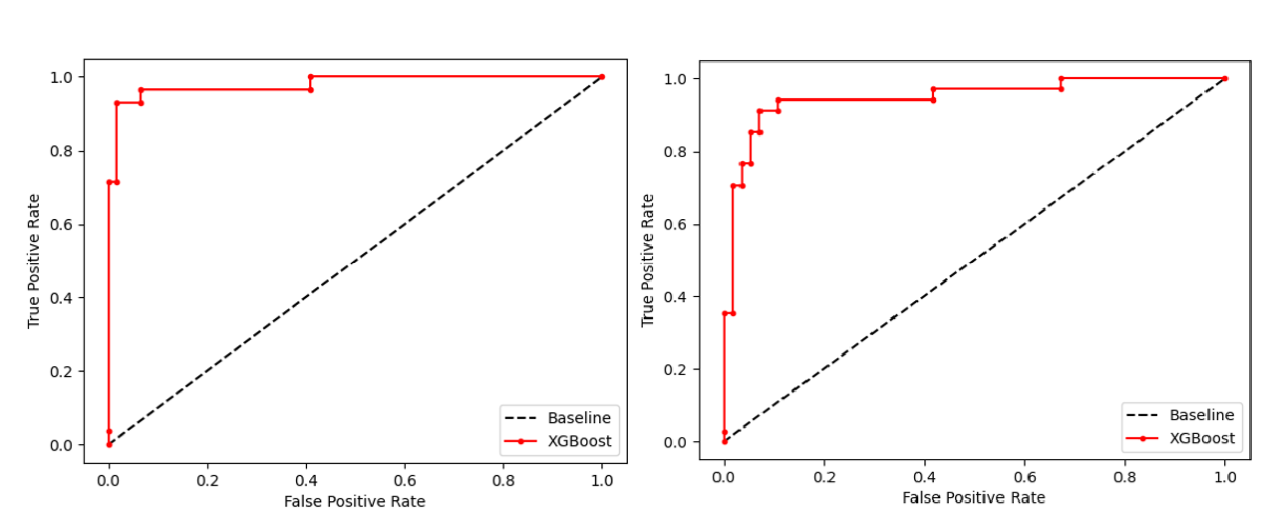 |
| **Figure S6**. Receiver Operating Characteristic (ROC) curves for XGBoost models including outcomes coded by the AND (left) and OR (right) rules and three sets of geometric descriptors used as independent variables. Top figures: Models including all geometric descriptors. Middle figures: Models including a subset of geometric descriptors chosen by the Recursive Elimination algorithm. Bottom figures: Models including a subset of geometric descriptors chosen by the Boruta algorithm. |
| 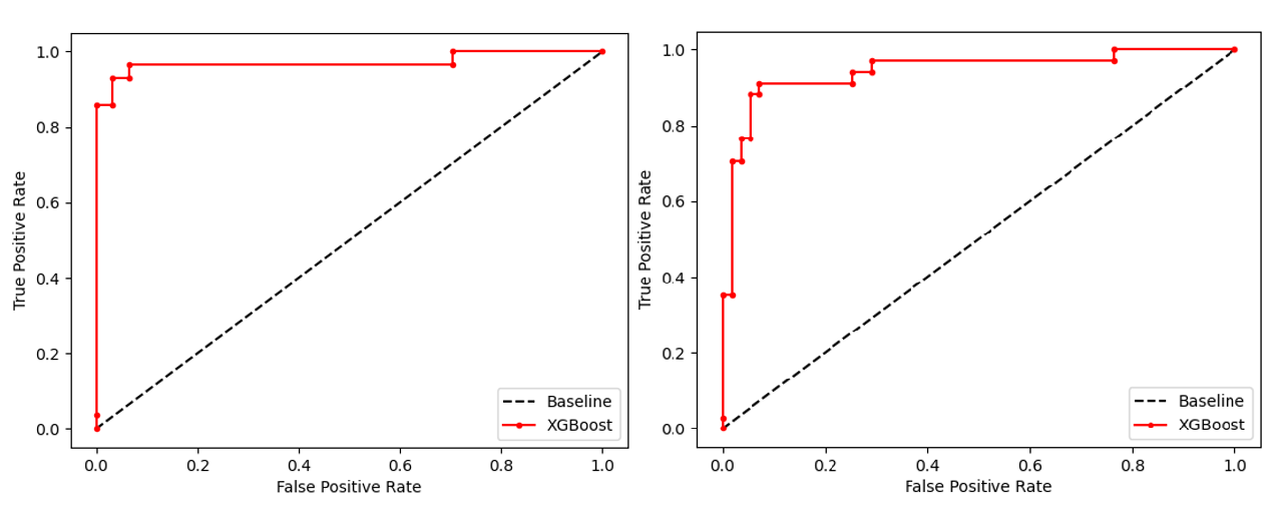 |
| 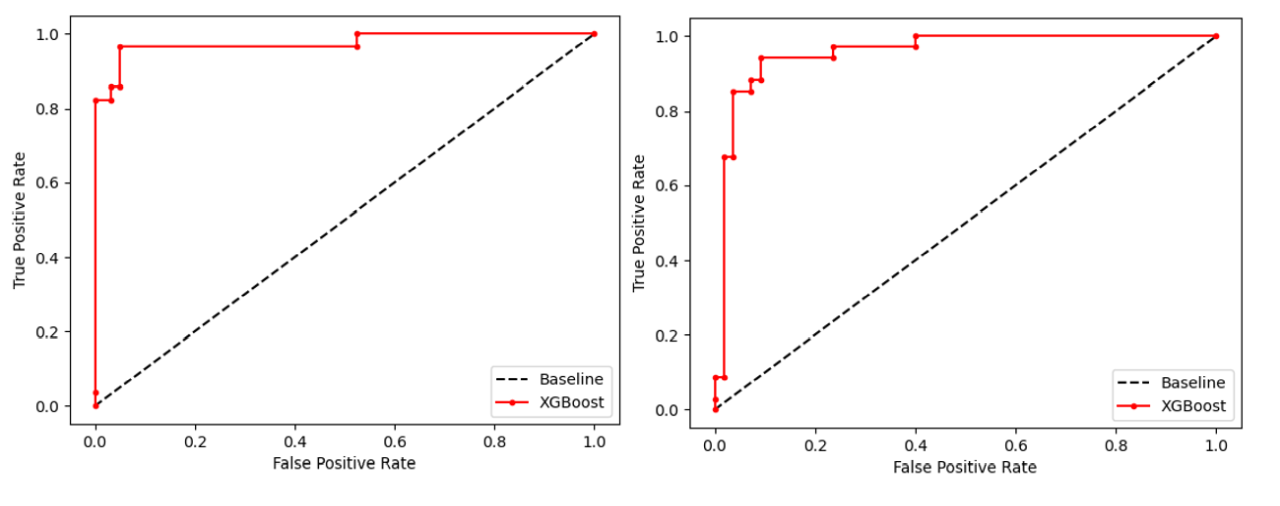 |
| 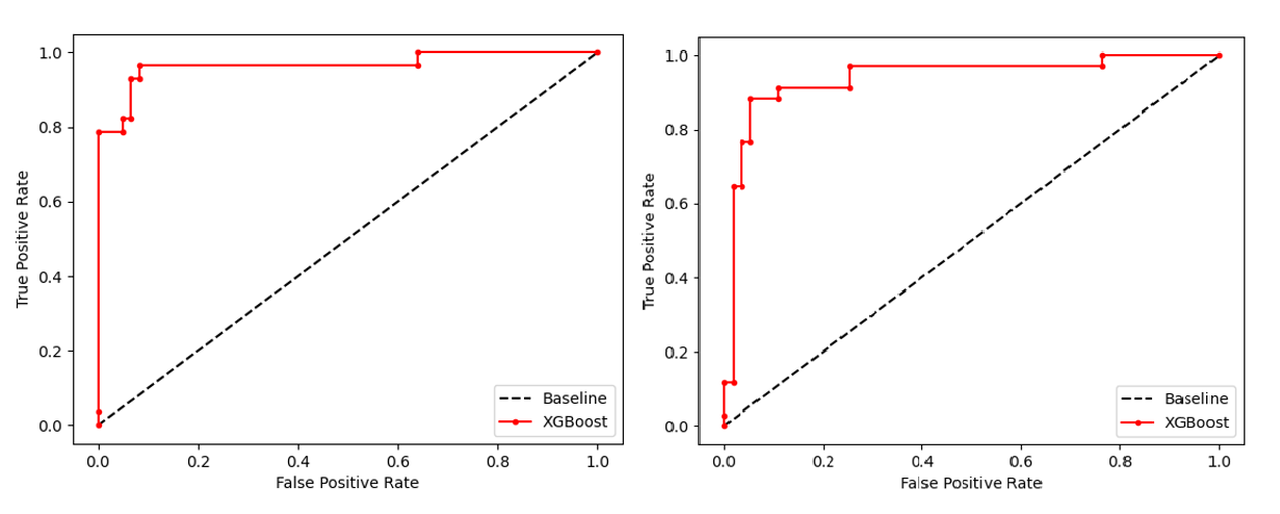 |
| **Figure S7**. Receiver Operating Characteristic (ROC) curves for XGBoost models including outcomes coded by the AND (left) and OR (right) rules and three sets of geometric descriptors used as independent variables. Top figures: subsets excluding the geometric descriptors for ‘whiskers change’. Middle figures: subsets excluding the geometric descriptos for ‘head position’. Bottom figures: subsets excluding geometric descriptors for both action units (whiskers change and head position). |


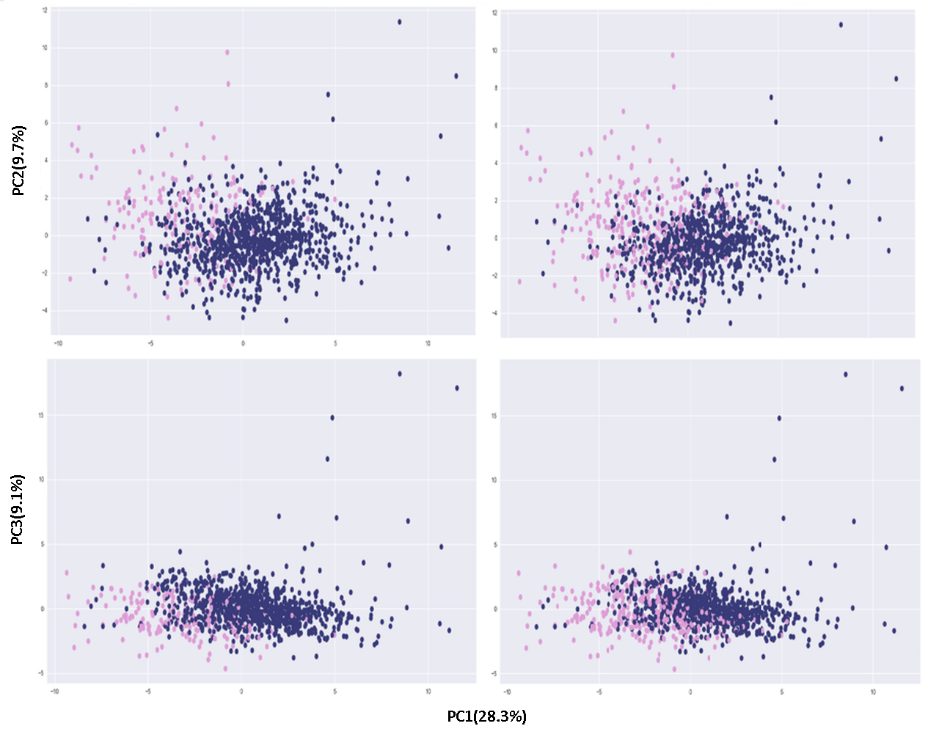


**Figure S8.** Principal component analysis performed on the matrix of standardized geometric descriptors. The cats were classified as painful (lilac) and non-painful (dark blue) using the AND_rule (left figures) and OR_rule (right figures). Top figures: Second (PC2) vs first principal components (PC1). Bottom figures: Third (PC3) vs first principal component (PC1).


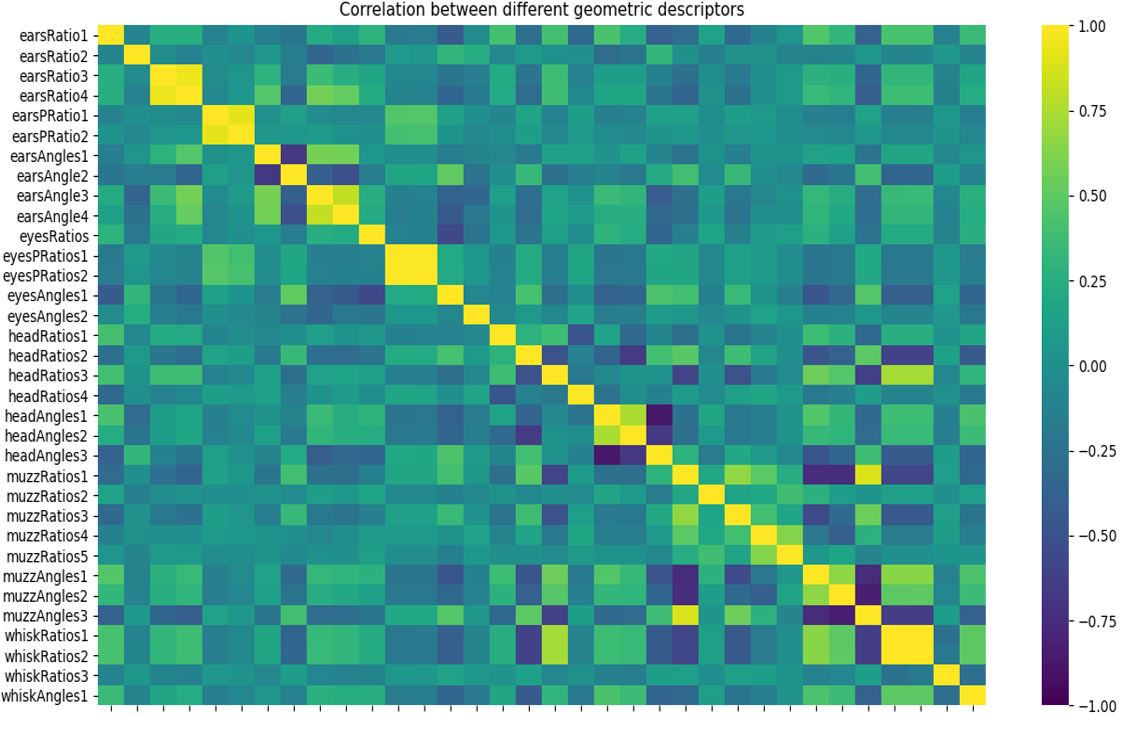


**Figure S9**. Plot of Principal Component Analysis (PCA) depicting the correlation matrix for 35 geometric descriptors.

**Supplementary information – Landmark description**


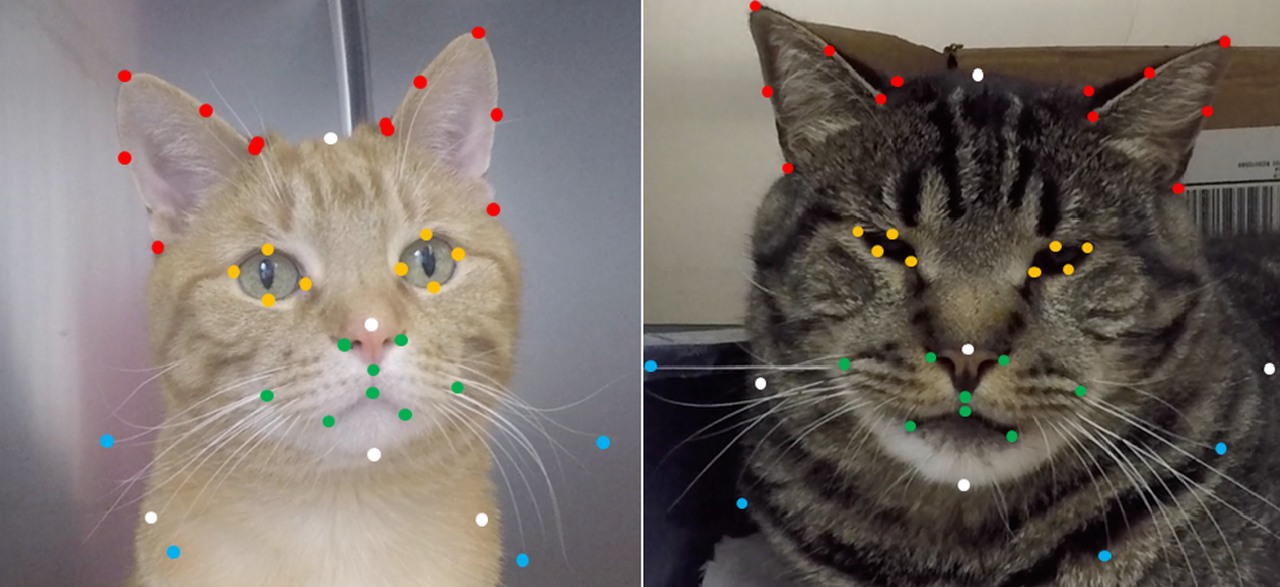


**Figure S10.** Examples of images used to define facial landmarks based on the five action units of the Feline Grimace Scale^©^ (Ear position: red; Orbital tightening: yellow; Muzzle tension; green; Whiskers change: blue; Head position: white). Left: non-painful cat (FGS score = 0). Right: painful cat (FGS score = 9).


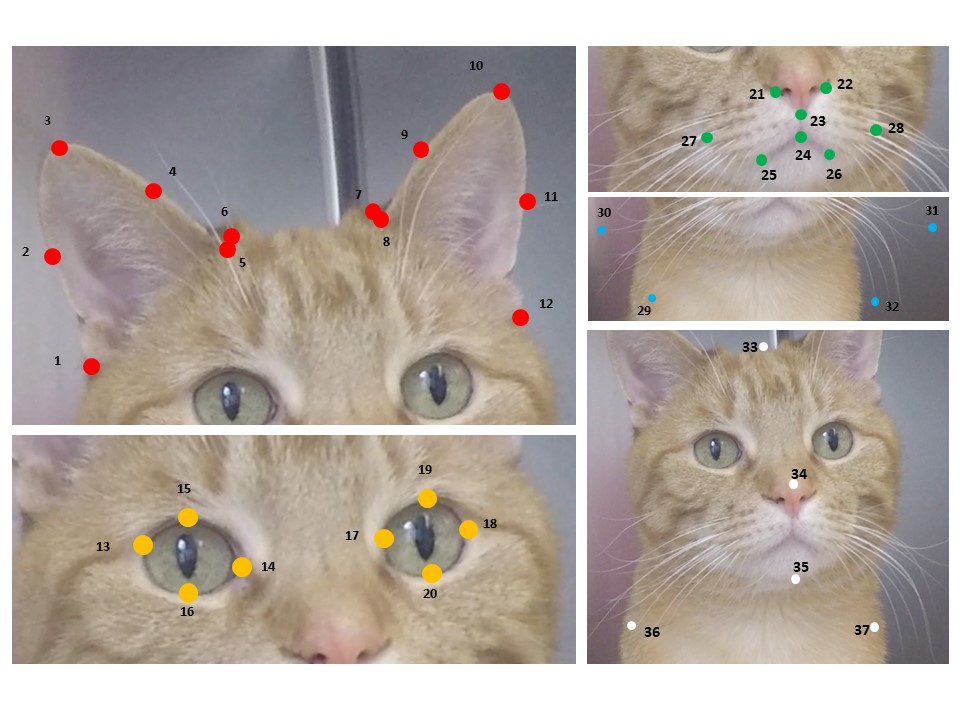


**Figure S11.** Close-up of a cat’s face with numbered facial landmarks delineating each action unit of the Feline Grimace Scale^©^ (Ear position: red; Orbital tightening: yellow; Muzzle tension; green; Whiskers change: blue; Head position: white).

**Table S1** – Anatomical description of facial landmark positions based on the action units of the Feline Grimace Scale^©^ (FGS).

| **Action Unit (FGS)** | **Landmark number** | **Description** |
| --- | --- | --- |
| **Ear position** | 1 | right ear; ventrolateral border; point connecting the ear and the head |
|  | 2 | right ear; lateral border; midway between the tip of the ear and the point connecting the ear and the head |
|  | 3 | right ear; tip of the ear |
|  | 4 | right ear; medial border; midway between the tip of the ear and the point connecting the ear and the head |
|  | 5 | right ear; medial border; rostral point connecting the ear and the head |
|  | 6 | right ear; medial border; caudal point connecting the ear and the head  Obs: when the ears are in neutral position, landmarks 5 and 6 may be overlapping |
|  | 7 | left ear; medial border; caudal point connecting the ear and the head  Obs: when the ears are in neutral position, landmarks 7 and 8 may be overlapping |
|  | 8 | left ear; medial border; rostral point connecting the ear and the head |
|  | 9 | left ear; medial border; midway between the tip of the ear and the point connecting the ear and the head |
|  | 10 | left ear; tip of the ear |
|  | 11 | left ear; lateral border; midway between the tip of the ear and the point connecting the ear and the head |
|  | 12 | left ear; ventrolateral border; point connecting the ear and the head |
| **Orbital tightening** | 13 | right eye; lateral canthus |
|  | 14 | right eye; medial canthus |
|  | 15 | right eye; dorsal margin |
|  | 16 | right eye; ventral margin |
|  | 17 | left eye; medial canthus |
|  | 18 | left eye; lateral canthus |
|  | 19 | left eye; dorsal margin |
|  | 20 | left eye; ventral margin |
| **Muzzle tension** | 21 | lateral aspect of the right nares |
|  | 22 | lateral aspect of the left nares |
|  | 23 | midline of the ventral tip of the nose |
|  | 24 | commissure of the lips (midline) |
|  | 25 | right lateral commissure of the lips |
|  | 26 | left lateral commissure of the lips |
|  | 27 | right lateral border of the muzzle |
|  | 28 | left lateral border of the muzzle |
| **Whiskers change** | 29 | right whiskers; the most ventrally visible whisker originating from the muzzle  Obs: imagine a triangle including points 29, 30 and a point in the middle of the muzzle that includes most whiskers |
|  | 30 | right whiskers; the most dorsally visible whisker originating from the muzzle |
|  | 31 | left whiskers; the most dorsally visible whisker originating from the muzzle  Obs: imagine a triangle including points 31, 32 and a point in the middle of the muzzle that includes most whiskers |
|  | 32 | left whiskers; the most ventrally visible whisker originating from the muzzle |
| **Head position** | 33 | top of the head (midline) |
|  | 34 | top of the nose (midline) |
|  | 35 | ventral chin (midline) |
|  | 36 | imaginary point at the right scapula |
|  | 37 | imaginary point at the left scapula  Obs: landmarks 36 and 37 are approximately on the same horizontal line |

**Supplementary information – Geometric descriptors**

**Notation used in all geometric descriptors**

da-b: Euclidean distance between landmarks a and b

$A_{∷(a,b,c,d)}$ : area of the quadrilateral whose vertices are landmarks a, b, c and d

$∡\left( a,b,c \right)$ : angle formed by the lines a-b and a-c

The scores defined for each of the 5 action units (AU) are presented in separate sections below.

####

#### **Ear position:** 10 geometrical scores are linked to this AU (Figure 1).

$\mathrm{eRatio}1=\frac{1}{2}\left( \frac{d_{5-6}}{d_{5-8}}+\frac{d_{8-7}}{d_{8-5}} \right)$

$\mathrm{eRatio}2=\frac{1}{3}\left( \frac{d_{3-10}}{d_{5-8}}+\frac{d_{2-11}}{d_{5-8}}+\frac{d_{4-9}}{d_{1-12}} \right)$

$\mathrm{eRatio}3=\frac{1}{2}\left( \frac{d_{1-10}}{d_{19-14}}+\frac{d_{12-3}}{d_{19-14}} \right)$

$\mathrm{eRatio}4=\frac{1}{2}\left( \frac{d_{2-10}}{d_{19-14}}+\frac{d_{11-3}}{d_{19-14}} \right)$

$\mathrm{ePRatio}1=\frac{A_{∷(4,5,8,9)}}{A_{∷(1,12,18,13)}}$

$\mathrm{ePRatio}2=\frac{A_{∷(5,6,7,8)}}{A_{∷(1,12,18,13)}}$

$$\mathrm{eAngle}1=\frac{1}{2}\left( ∡\left( 5,3,8 \right)+∡\left( 8,5,10 \right) \right)$$

$\mathrm{eAngle}2=\frac{1}{2}\left( ∡\left( 5,1,3 \right)+∡\left( 8,12,10 \right) \right)$

$\mathrm{eAngle}3=\frac{1}{2}\left( ∡\left( 1,3,12 \right)+∡\left( 12,10,1 \right) \right)$

$\mathrm{eAngle}4=\frac{1}{2}\left( ∡\left( 1,2,11 \right)+∡\left( 12,11,2 \right) \right)$

| **Figure S12**: Ear position. Left: angles for Ear position: eAngles2, eAngles3, eAngles4 and eAngles1. Top right: Quadrilaterals and landmarks used for the calculation of ratios: ePRatio1 y ePRatio2. Top left: Lines indicating landmarks used to calculate distance ratios: eRatios1, eRatios2, eRatios3 y eRatios4. |
| --- |


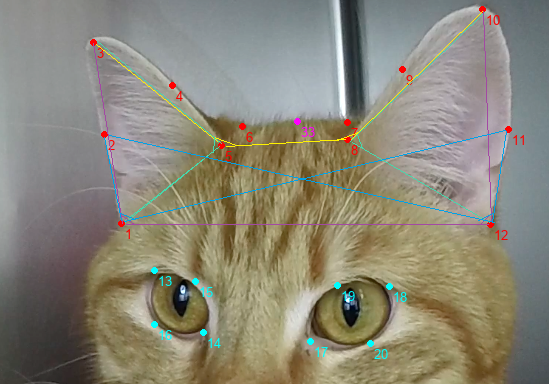

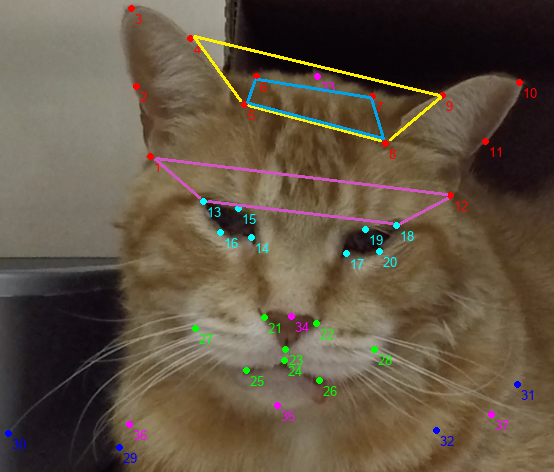

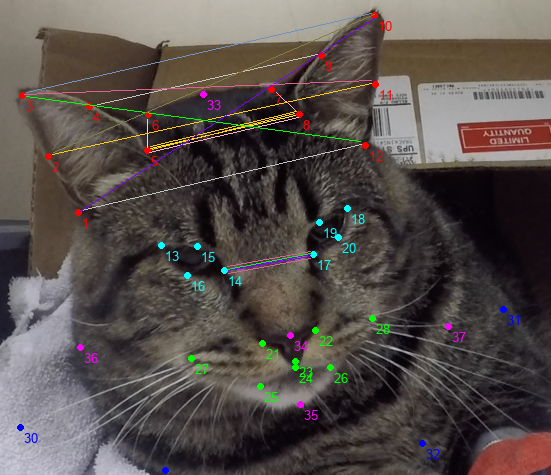


#### **Orbital tightening**: 5 geometrical scores are linked to this AU (Figure 2).

$eyRatio0=\frac{1}{2}\left( \frac{d_{13-14}}{d_{15-16}}+\frac{d_{17-18}}{d_{19-20}} \right)$

$eyAngle1=\frac{1}{2}\left( ∡\left( 14,15,16 \right)+∡\left( 17,19,20 \right)+∡\left( 13,15,16 \right)+∡\left( 20,18,19 \right) \right)$

$eyAngle2=\frac{1}{2}\left( ∡\left( 15,14,16 \right)+∡\left( 18,17,19 \right) \right)$

$eyPRatio1=\frac{1}{2}\left( \frac{A_{∷(13,15,14,16)}}{A_{∷(5,8,18,13)}}+\frac{A_{∷(17,18,19,20)}}{A_{∷(5,8,18,13)}} \right)$

$eyPRatio2=\frac{A_{∷(15,19,20,16)}}{A_{∷(5,8,18,13)}}$


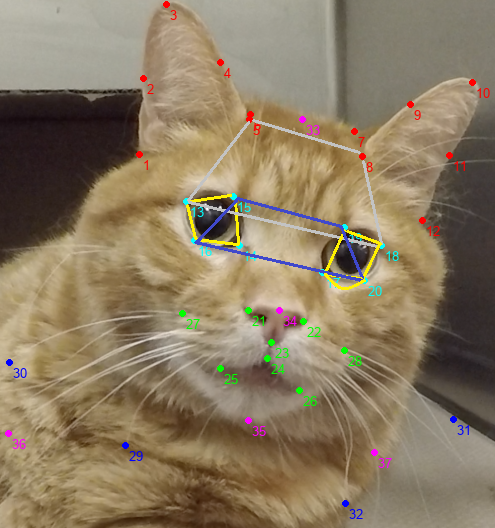


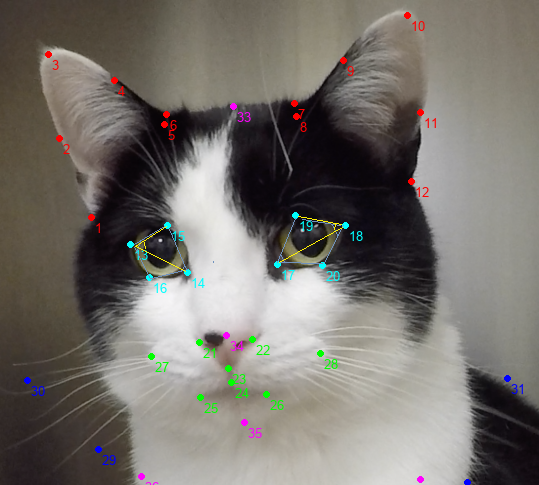

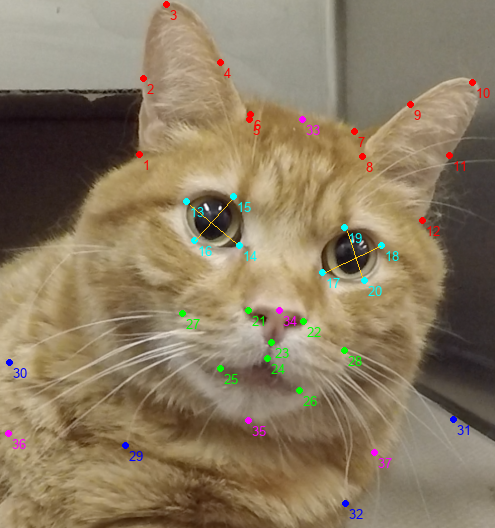


| **Figure S13:** Orbital tightening. Left: Lines between facial landmarks indicating the calculated angles: eyAngles1 and eyAngles2. Top right: Quadrilaterals and landmarks used for the calculation of ratios: eyPRatio1 y eyPRatio2. Top left: Lines indicating landmarks used to calculate distance ratio: eRatios0. |
| --- |

**Muzzle tension:** 8 geometrical scores are linked to this AU (Figure 3).

$mRatio1=\frac{1}{2}\left( \frac{d_{21-25}}{d_{21-22}}+\frac{d_{26-22}}{d_{21-22}} \right)$

$mRatio2=\frac{1}{2}\left( \frac{d_{27-23}}{d_{21-22}}+\frac{d_{28-23}}{d_{21-22}} \right)$

$mRatio3=\frac{d_{23-24}}{d_{21-22}}$

$mRatio4=\frac{1}{2}\left( \frac{d_{25-23}}{d_{23-22}}+\frac{d_{26-23}}{d_{23-22}} \right)$

$mRatio5=\frac{d_{25-26}}{d_{23-24}}$

$mAngle1=\frac{1}{2}\left( ∡\left( 21,24,27 \right)+∡\left( 22,24,28 \right) \right)$

$mAngle2=∡\left( 24,25,26 \right)$

$$mAngle3=\frac{1}{2}\left( ∡\left( 24,21,25 \right)+∡\left( 24,22, 26 \right) \right)$$

| **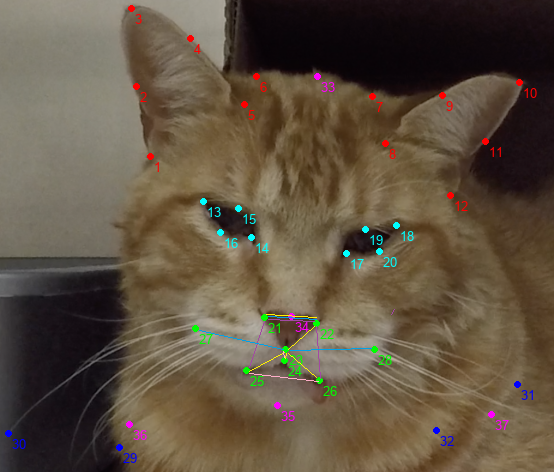** | **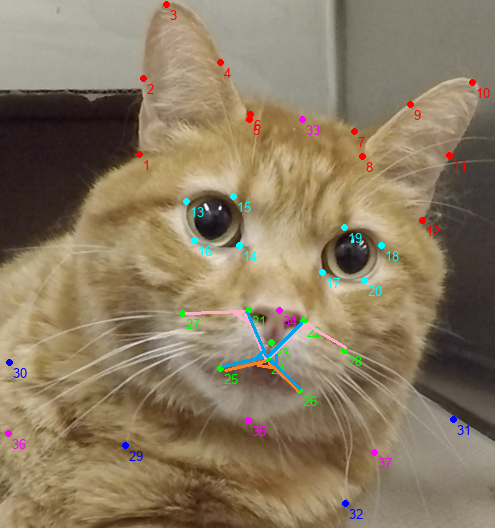** |
| --- | --- |
| **Figure S14:** Muzzle tension. Left: Lines indicating landmarks used to calculate distance ratios: mRatio1, mRatio2, mRatio3, mRatio4 and mRatio5. Right: Angles calculated to determine the muzzle tension: mAngle1, mAngle2 and mAngle3. | |

####

#### **Whiskers change**: 5 geometrical scores are linked to this AU (Figure 4).

$wRatio1=\frac{1}{2}\left( \frac{d_{30-31}}{d_{34-35}}+\frac{d_{29-32}}{d_{34-35}} \right)$

$wRatio2=\frac{1}{2}\left( \frac{d_{29-31}}{d_{34-35}}+\frac{d_{30-32}}{d_{34-35}} \right)$

$wAngle1=\frac{1}{2}\left( ∡\left( 34,30,29 \right)+∡\left( 34,31,32 \right) \right)$

$wAngle2=\frac{1}{2}\left( ∡\left( 34,30,31 \right)+∡\left( 34,29,32 \right) \right)$

$wAngle3=\frac{1}{2}\left( ∡\left( 35,30,31 \right)+∡\left( 35,29,32 \right) \right)$

| 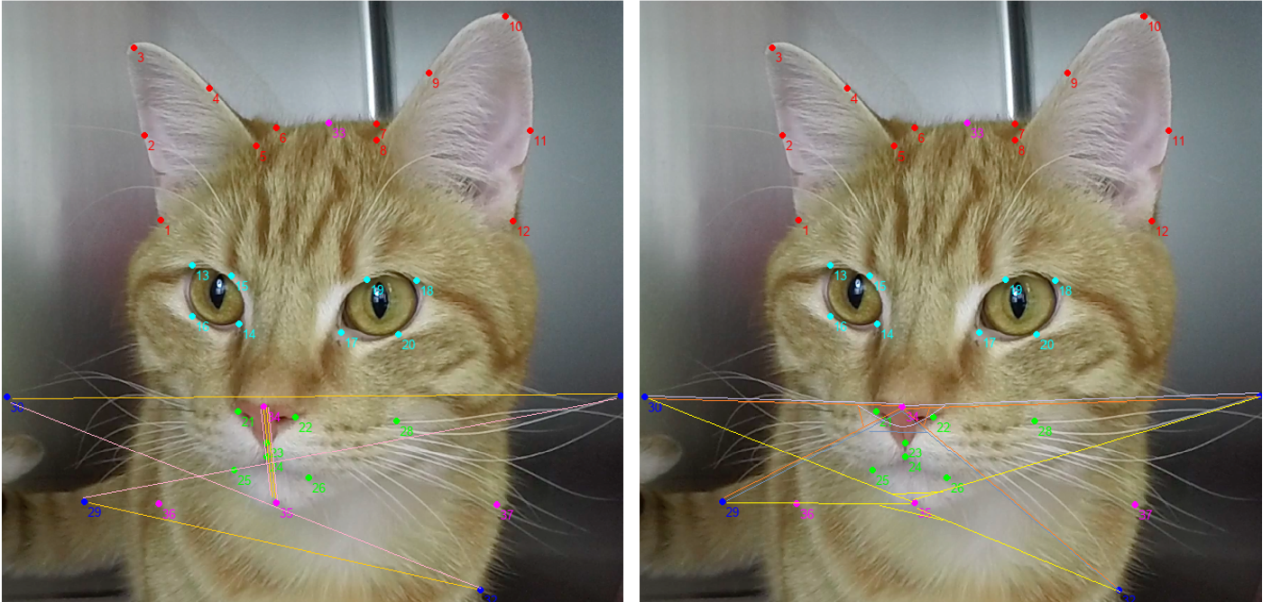 |
| --- |
| **Figure S15**: Left: Whisker change. Left: Lines indicating landmarks used to calculate distance ratios: wRatio1 and wRatio2. Right: Angles for whiskers change: wAngle1, wAngle2 and wAngle3. |

#### **Head position:** 7 geometrical scores are linked to this AU (Figure 5).

$hRatio1=\frac{d_{34-33}}{d_{36-37}}$

$hRatio2=\frac{d_{34-35}}{d_{36-37}}$

$hRatio3=\frac{1}{2}\left( \frac{d_{37-33}}{d_{34-35}}+\frac{d_{36-33}}{d_{34-35}} \right)$

$hRatio4=\frac{1}{2}\left( \frac{d_{35-37}}{d_{35-33}}+\frac{d_{36-35}}{d_{35-33}} \right)$

$hAngle1=\frac{1}{2}\left( ∡\left( 37,35,33 \right)+∡\left( 36,35,33 \right) \right)$

$hAngle2=\frac{1}{2}\left( ∡\left( 33,36,37 \right)+∡\left( 34,36,37 \right) \right)$

$hAngle3=\frac{1}{2}\left( ∡\left( 34,33,37 \right)+∡\left( 34,33,36 \right) \right)$

| 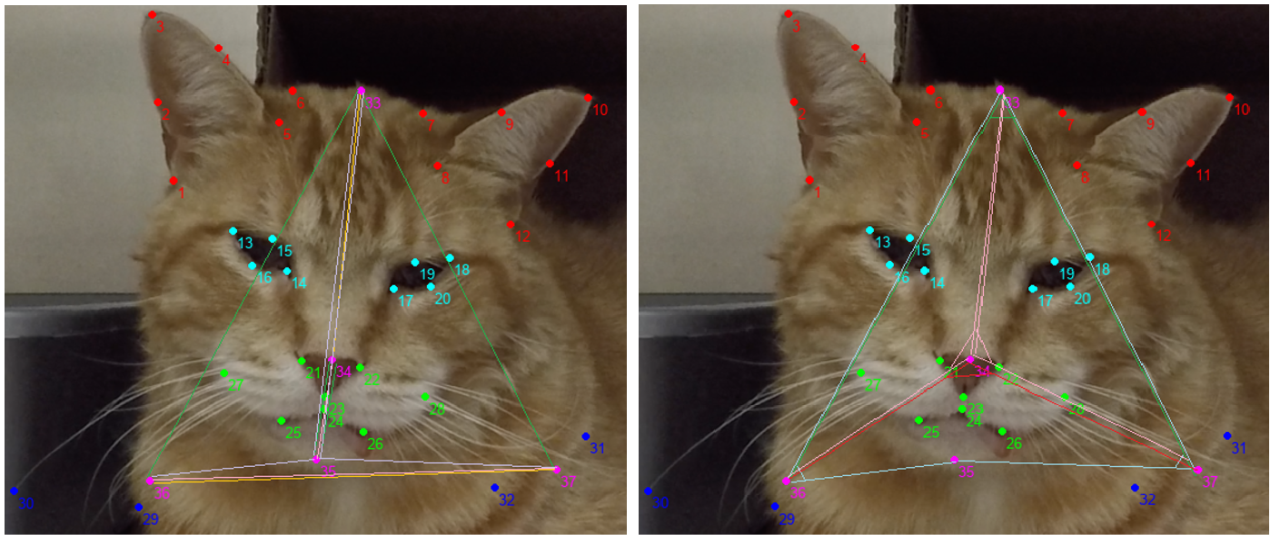 |
| --- |
| **Figure S16**: Left: Head position. Left: Lines indicating landmarks used to calculate distance ratios: wRatio1 and wRatio2. Right: Angles for head position: wAngle1, wAngle2 and wAngle3. |
